# Intake of Animal Protein and Dietary Sources in the Colombian Population: Results of the National Nutrition Survey (ENSIN-2015)

**DOI:** 10.1155/2022/2345400

**Published:** 2022-01-13

**Authors:** Oscar F. Herrán, María del Pila Zea

**Affiliations:** ^1^Escuela de Nutrición y Dietética, Universidad Industrial de Santander, Carrera 32 No. 29–31, Bucaramanga, Santander, Colombia; ^2^Universidad Javeriana de Cali, Facultad de Ciencias de la Salud, Calle 17 # 121B-80, Cali, Valle del Cauca, Colombia

## Abstract

**Background:**

There is a lack of knowledge in Colombia about dietary intake and sources of animal protein.

**Design:**

Cross-sectional, nationally representative surveys. *Setting*. Colombia. *Participants*. *n* = 32,457 participants aged from 1 to 64 years. The sample analyzed included 21,036 boys and nonpregnant girls, 10,099 adults, and 1,322 pregnant women, 118 of whom were below 18 years of age.

**Results:**

Protein intake was 32.9 g/d (95% CI: 32.4, 33.4) per 1,000 kilocalories. The relative contribution (%) of total protein to the total energy intake/day (acceptable macronutrient distribution) was 13.2% (95% CI: 13.0, 13.3). The acceptable macronutrient distribution (AMDR) for animal protein for those aged 1 to 64 years was 7.8% (95% CI: 7.6, 8.0), with a minimum of 7.1% (95% CI: 6.7, 7.5), which was for children aged from 13 to 17 years, and a maximum of 8.3% (95% CI: 8.1, 8.5), for children aged from 1 to 4 years (*P*=0.018). For all groups, animal protein made up the majority of total proteins, with 62.6% (95% CI: 61.7, 63.6) for preschoolers, 55.8% (95% CI: 53.2, 58.4) for school-aged children, 54.6% (95% CI: 53.0, 56.1) for adolescents, 58.1% (95% CI: 57.5, 58.7) for adults, and 57.5% (95% CI: 55.2, 59.7) for pregnant women (*P*=0.027). The three main dietary sources of animal protein were red meat (17.8%), chicken (16.3%), and eggs (10.5%). The sources of vegetal protein were bread-arepa-pasta (20.0%), cereals (19.8%), and legumes (8.2%).

**Conclusions:**

Protein intake is excessive according to the Recommended Dietary Allowance (RDA), while it is not excessive from the perspective of the AMDR.

## 1. Introduction

The importance of protein for the human diet is indisputable [1–4]. The quantity consumed (g), its animal or vegetable origin, and the dietary sources are synonymous with its quality [2]. The U.S. Institute of Medicine's recommended daily allowance for protein is 1.05 gram per kilogram per day (g/kg/day) for children aged from 1 to 3 years, 0.95 g/kg/d for children aged from 4 to13 years (19 g/d for girls and 34 g/d for boys), 0.85 g/kg/d for adolescents aged from 14 to 18 years (52 g/d for males and 46 g/d for females), and 0.8 g/kg/d for adults. For pregnant women, the RDA is 1.1 g/kg/d or 25 g/d in addition to the amount recommended for women of the same age [1]. Nevertheless, if instead of the RDA the acceptable macronutrient distribution range (AMDR) is used, in which protein accounts for 10 to 35% of the total energy/day, then the RDA would range from 1.05 to 3.67 g/kg/d [5].

The effect of excessive protein intake on the risk of death from all causes is controversial [6–8]. Protein intake from vegetable sources slightly reduces the overall risk of death and death from cardiovascular diseases (CVD) but not from cancer. In addition, the intake of total protein and protein from animal sources has not been found to be associated with death from all causes or from cardiovascular diseases or cancer [8]. The association of animal protein intake with mortality from CVD can be positive when protein intake is high relative to total energy and subjects present at least one risk factor related to lifestyle [7]. Animal protein intake (red meat and processed meat) can increase the risk of mortality from any cause and from CVD, while the consumption of red meat is positively associated with mortality from CVD [9, 10]. Higher consumption of vegetable protein and lower consumption of animal protein have been associated with a lower risk of metabolic syndrome. [11]. In the United States, according to the 2007–2010 National Health and Nutrition Examination Survey (NHANES), 62% of total protein was from animal sources (16% from milk and milk by-products and 46% from meat, chicken, eggs, and fish) and 30% from vegetable sources. In adults aged 19 years or older, mean animal protein intake ranged from 0.33 to 0.97 (g/kg/d) [3]. Based on the analysis of nine NHANES cycles (1999–2016), in the population above 20 years of age, protein intake and the relative contribution of protein to total energy increased from 15.5% to 16.4%, and said increase was due to both the consumption of protein of animal origin (0.44%) and vegetable (0.33%) sources (*P* < 0.001 for trends) [2]. Knowledge about whether protein intake from animal sources is predominant is lacking in Colombia, as it is in developed and middle-income countries. The dietary sources are also not known.

This study was based on data gathered from the 2015 National Nutrition Survey (ENSIN in Spanish). Its objectives included the following: (*a*) estimating total protein intake and animal and vegetable protein intake in the Colombian population aged from 1 to 64 years and for pregnant women, (*b*) relating this consumption to biological and sociodemographic variables, (*c*) describing the intake of total protein and animal and vegetable protein as AMDR, and, lastly, (*d*) determining the nutritional sources and their relative contribution (%) to total protein intake and to animal and vegetable intake.

## 2. Methods

In Colombia, South America, the Colombian Institute for Family Wellbeing (ICBF in Spanish) along with the Ministry of Health (MinSalud) conducted the ENSIN-2015, which is representative of 99% of the Colombian population [12]. The ENSIN-2015 estimated dietary intake using the 24-hour recall method (24 HR) [13, 14]. A total of 44,202 households were surveyed, representing 4,739 groups of 295 strata. The methods, study populations, scope, and limitations of the ENSIN-2015 have been previously published [12]. The target population for this analysis was children aged from 1 to 17 years, adults aged between 18 and 64 years, and pregnant girls and women. The ENSIN-2015 included 151,343 people. Protein intake was estimated in a weighted random subsample (*n* = 32,457). The sample analyzed included 21,036 boys and nonpregnant girls, 10,099 adults, and 1,322 pregnant women, 118 of whom were below 18 years of age. Of the total, 749 answered the second 24 HR.

### 2.1. Data Sources

The main output variables were the following: (a) total, animal, and vegetable protein intake, in grams/day (g/d) and also expressed as grams per 1,000 kilocalories consumed [15, 16] (proteins (g/d) ∗ 1000/total kilocalories/day) (g/1000/d) and (b) the relative contribution or AMDR (%) of food items to total intake and animal and protein intake [5]. The 24 HR was based on the methodology developed in 1999 by the U.S. Department of Agriculture (USDA) [14, 17, 18]. In brief, this consisted of asking about dietary consumption over the last 24 hours, ensuring the completion of what is known as the Automated Multiple-Pass Method [14, 17, 18]. The 24 HR had a response rate of 92%. After rigorous data cleaning of the database, which was obtained from an electronic application designed to capture 24 HR data gathered from the interview, the correct coding of the foods and preparations was ensured based on a food composition table (FCT). The FCT used in the ENSIN-2015 shows the composition of 2,703 items in 100 g of the edible portion. The authors supplemented the FCT with the percentage of animal protein in each item based on their experience and standard recipes, when necessary. After calculating the amount (g) of animal protein in the FCT [total protein (g) ∗ % estimated animal protein/100], the amount (g) of animal protein in 100 g of the item in the FCT was obtained. The amount of vegetable protein was calculated as (total protein (g) − animal protein (g)). FoodCalc® v1.3 [19] was used to translate the intake recorded in the 24 HR into energy/day (kilocalories/day) and grams of total protein, animal protein, and vegetable protein. In addition, the relative contribution (%) of animal and vegetable protein to total protein and the relative contribution (%) of protein intake to total energy were calculated. That was based on [(total protein (g/d) ∗ 4/total kilocalories ingested/day) ∗ 100]. Lastly, the amount of total protein and protein from animal and vegetable sources per kilogram of actual weight and “adequate weight” (g/kg/d) was calculated. The ENSIN-2015 calculated “adequate weight” for each individual who was underweight or overweight based on two predictive equations, one for subjects aged from 1 to 17 years and another for subjects aged from 18 to 64 years. The equations incorporated data on sex, weight, size, and age of individuals with normal nutritional status according to age group. The relative contribution (%) of the food items to total protein and protein from animal and vegetable sources was calculated as ((total protein per item/total protein) ∗ 100), ((animal protein per item/total animal protein) ∗ 100), and ((vegetable protein per item/total vegetable protein) ∗ 100), respectively.

Nutritionists and trained staff administered the surveys to obtain sociodemographic data on the households and biological data from individuals. Household food insecurity was determined using the Latin America and Caribbean Scale (ELCSA in Spanish) adopted by the United Nations Food and Agriculture Organization (FAO) [20]. This 15-question scale is based on the perception of uncertainty and concern about access to food and the experience of a reduced amount of food. The ELCSA classifies households as secure and insecure and the level of food insecurity as low, moderate, and severe [20]. The wealth index is a continuous indicator of the socioeconomic level of the household and was categorized by quartiles. It was determined in the total of sample of the survey (ENSIN-2015) based on a principal components analysis of the sets of physical household characteristics and goods and services available, all predetermined by international demographic and health surveys [21]. Educational level of the head of household was determined based on years of study with a passing grade. The geographic region is a variable that represents the country and its degree of structural development, economic conditions, and the culture of the subjects. Colombia has 5 geographic regions [12, 22], where Bogotá is the capital of the country and has the highest human development index, with the central region. The Pacific and the Amazonia-Orinoquia regions are the poorest. In terms of race, the population of the Pacific region is predominantly Black, and that of the Amazonia-Orinoquia region is predominantly indigenous and mestizo [23]. The degree of urbanism was categorized according to three categories based on population density: urban centers and large cities with over 1 million residents, small towns with 100,000 ≥ 1 million residents, and dispersed populations with less than 100,000 residents [12].

Anthropometric measurements were obtained from all household members using well-known standard techniques and calibrated instruments [24]. The ENSIN-2015 used stadiometers to measure height (ShorrBoard) with a sensitivity of 1 mm and digital scales (SECA 874) to measure weight with a precision of 100 g. For children, *Z*-scores were determined for height-for-age and body mass index (BMI), in accordance with growth standards by the World Health Organization (WHO) [25].

### 2.2. Data Analysis

The distributions of kilocalorie and protein intake were normalized and corrected to incorporate intraperson variability using methods proposed by the University of Iowa, with PC-Side v1.0 software [26]. All the analyses incorporated the effect of the complex sampling design with Stata v14.1 [27]. The analysis was aimed at (a) describing the intake of total protein and animal and vegetable protein adjusted by energy density (g/1000/d) for the categories of the socioeconomic variables, (b) establishing crude and adjusted differences in animal and vegetable protein intake (g/1000/d) for the categories of the variables studied, using simple and multiple linear regression models with protein intake (g/1000/d) as the dependent variable and the covariables as explanatory variables, (c) determining the relative contribution (%) of total protein intake to total energy/day (kilocalories/day), (d) establishing the relative contribution (%) of animal and vegetable protein to total protein/day, (e) establishing the relative contribution (%) of food items to total protein and animal and vegetable protein intake by age group and for pregnant women, and (f) determining the amount of total protein (g/d) and animal and vegetable protein (g/1000/d) per kilo of actual weight and “adequate” weight. The description was performed with averages or proportions ± the standard error (SE) and 95% confidence intervals (95% CI).

This study was conducted in accordance with the guidelines set forth by the Declaration of Helsinki [28]. Consent for participation in the study was obtained by the Colombian Institute for Family Wellbeing prior to inclusion [12, 29]. The health research ethics committee of Universidad Industrial de Santander determined that the analyses of these anonymized data were exempt from review.

## 3. Results

Mean protein intake by the Colombian population aged from 1 to 64 years is 32.9 g/1000/d (95% CI: 32.4, 33.4), animal protein intake is 19.4 g/1000/d (95% CI: 18.9, 19.8), and vegetable protein is 13.9 g/1000/d (95% CI: 13.5, 14.2) (Table 1). The relative contribution of animal protein to total energy in the study population (age from 1 to 64 years) is 7.8% (95% CI: 7.6, 8.0), with a minimum of 7.1% (95% CI: 6.7, 7.5) for those aged from 13 to 17 years and a maximum of 8.3% (95% CI: 8.1, 8.5) for children aged from 1 to 4 years, *P*=0.018 (Tables 1S–5S). Table 1 presents the consumption details for each sociodemographic variable. Animal protein intake is positively associated with wealth level and region. Intake is 2.5 g/1000/d greater in Bogotá, the capital of the country, than in the central region, and it is lowest in the Pacific, which is the poorest region, *P*=0.002 (Table 2). More vegetable protein is consumed in the five main cities than in the rest of the cities, *P*=0.026 (Table 3). No differences were found in animal or vegetable protein intake (*P* ≥ 0.05) among any of the categories related to age group, nutritional status, food security, or education of head of household. For children aged from 5 to 12 years, females consume more animal protein than males, *P*=0.031 (Table 2).

**Figure 1:**
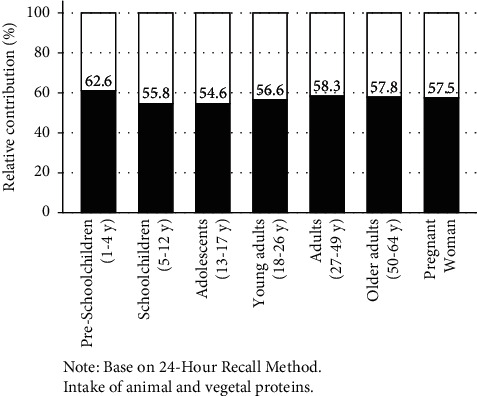
Relative contribution (%) to total protein intake in the Colombian population. National Survey of Nutritional Situation in Colombia (ENSIN-2015). Animal protein (black); 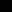
vegetal protein (white) 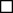

In the overall population aged from 1 to 64 years, the three main sources of total animal protein intake are, in order, meat (17.8%), chicken (16.3%), and eggs (10.5%). The three main sources of total vegetable protein intake are bread-arepa-pasta (20%, primarily wheat and corn), cereals (19.8%, mainly rice), and legumes (8.2%) (Table 4).  Table 6S shows the contribution (%) of dietary sources to protein intake by age group. The relative contribution of animal protein to total protein is 56.6% (95% CI: 55.1, 58.0). Animal protein makes up the majority of the protein intake for all age groups: 62.6% (95% CI: 61.7, 63.6) for preschoolers, 55.8% (95% CI: 53.2, 58.4) for school-aged children, 54.6% (95% CI: 53.0, 56.1) for adolescents, 58.1% (95% CI: 57.5, 58.7) for adults, and 57.5% (95% CI: 55.2, 59.7) for pregnant women, *P*=0.027 (Tables 1S–5S and Figure 1).

For the overall population, the relative contribution (%) of total proteins to the total energy/day is 13.2% (95% CI: 13.0, 13.3), with a minimum of 12.7% (95% CI: 12.2, 13.2) for adolescents and a maximum of 13.3% (95% CI: 13.1, 13.6) for adults (Tables 1S–5S). These tables also show the mean total protein intake and mean animal and vegetable intake per age group and sex for the actual weight and the theoretically ideal weight. Milk is the main source of protein for children aged from 1 to 4 years (10.1%), and chicken (11.3%) and meat (11.2%) are the main sources for adolescents. For pregnant women, mean animal protein intake (average ± SD) is 20.9 ± 1.5 g/1000/d and mean vegetable protein intake is 14.3 ± 0.8 g/1000/d. Tables 7S–18S present the protein intake's details per age group and the relationships with some of the socioeconomic variables.

## 4. Discussion

In the Colombian population aged from 1 to 64 years, absolute protein intake ranges from 54.5 to 71.4 (g/d), and it ranges from 31.8 to 33.4 g/1000/d when adjusted for energy density. No differences were found between pregnant and nonpregnant women. The relative contribution (%) of animal protein to total protein/day is 54.6% to 62.6%. The children aged from 1 to 4 years present the greatest intake per kilogram of body weight, with 4.0 g/kg/d, as well as the greatest relative contribution (%) of animal protein to total protein/day, with 62.6%. For all age groups and pregnant women, the relative contribution (%) of total protein to energy intake is 13% to 14%. Protein intake was found to be positively associated with wealth level, the region, and the degree of urbanism. Animal protein intake is positively correlated with wealth level, which is higher in the country's capital, Bogotá, and in Orinoquía-Amazonía than in other regions. Vegetable protein intake is greater in the large cities than in the medium-sized cities and towns with a dispersed population. Meat, chicken, eggs, and milk make up 51.7% of the animal protein. Bread-arepa-pasta (mainly from wheat and corn), cereal (mainly rice), legumes, and potatoes make up 54.7% of the vegetable protein. These same food items make up 55% of the total protein in the diet.

In Colombia, the 2005 and ENSIN-2015 surveys [29, 30] have estimated dietary intake as well as total proteins over the past 20 years. Since the 2005 ENSIN did not estimate protein intake according to protein source, these results cannot be compared. Nevertheless, that survey reported that at least 36% of the Colombian population had a protein intake deficit associated with poverty [30]. A multicentric study performed from 2009 to 2011 with children between 1 and 17 years of age found total protein intake to be positively associated with wealth level and excessive protein intake to be associated with a body mass index >25 [31]. In that same study, the relative contribution (%) of total protein to total energy/day was 12.0% ± 0.11 [30]. The ENSIN-2015 also reported this finding [29]. In a cohort of children in Bogotá, it was found that those in the highest quartile of adherence to the consumption of a “protein” dietary pattern had fewer behavioral problems [32].

The comparison of our findings with other national surveys is limited. In the United States, the relative contribution of animal protein to total protein is 62% for adults aged 19 years or older according to the 2007–2010 NHANES [3] and 64.6% according to the 1999–2016 NHANES [2], compared to 58.1% in the present study for the same age group. The relative contribution of vegetable protein to total protein is 30% in the 2007–2010 NHANES [3], 35.1% in the 1999–2016 NHANES [2], and 43% in the present study (Table 5S). In absolute terms, women consumed less total protein than men in both the NHANES and the ENSIN-2015, but there were no significant dietary differences when adjusting by energy density and when considering intake relative to body weight. Nevertheless, the classification of the animal and vegetable sources of protein is not entirely comparable between the NHANES and the ENSIN-2015. Although absolute intake is not a good indicator, in the 2007–2010 NHANES [3], adults aged 19 years or older consumed more total protein per day on average (82.3 ± 0.8 (g/d)) than those in the ENSIN-2015 (67.3 ± 1.0 (g/d)). According to the French National Cross-Sectional Food Consumption Survey (INCA2; 2006-2007), [33] the average total protein intake for the adult population aged 18 years or older in France is 86.0 g/d ± 16, relative contribution to total energy/day is 16.8% ± 3.0, animal protein intake is 60.5 g/d, and vegetable protein intake is 25.5 g/d, while the relative contribution of animal protein to total protein intake is 69.5% ± 10.5 and the relative contribution of vegetable protein is 30.5% ± 10.5.

The relative contribution of animal protein to total energy/day is 7.9% ± 0.1 in the ENSIN-2015 and 11.9 ± 3.6 in the 2006-2007 INCA2, [33] while vegetable protein is 5.5 ± 0.0 in the ENSIN-2015 and 5.7 ± 0.1 in the 2006-2007 INCA2. According to estimates by FAOSTAT for 2014–2016, [34] total protein intake is 112 g/d in the United States, 104 g/d in Germany, 103 g/d in Argentina, 100 g/d in Canada, 97 g/d in Egypt, 92 g/d in Mexico, 87 g/d in Chile, 76 g/d in Peru, and 69 g/d in Colombia (4 g/d more than in 2005). Furthermore, for those same countries and period, animal protein intake (% of total protein) was 72 g/d (64%) in the United States, 62 g/d (60%) in Germany, 66 g/d (65%) in Argentina, 53 g/d (53%) in Canada, 24 g/d (24%) in Egypt, 43 g/d (47%) in Mexico, 28 g/d (51%) in Chile, 28 g/d (36.7) in Peru, and 36 g/d (52%) in Colombia [34].

In the 2007–2010 NHANES, the main sources of animal protein were, in order, cheese, chicken, low-fat milk, and cured and processed meat, as well as preparations with meat and eggs, which make up an average 75.1% of the total protein. The main sources of vegetable protein are wheat bread with yeast, rolls, nuts, and seeds, as well as preparations with pasta, which contribute an average 25.3% of total protein [3]. The increase in animal protein intake in the 1999–2016 NHANES is attributable to chicken (poultry) and eggs, and the increase in vegetable protein is attributable to the consumption of whole grains, seeds, and soybean [2]. In the 2006-2007 INCA2, the main sources of animal protein are, in order, red meat, poultry and game, viscera, and processed meat, milk, and milk by-products, which make up 58.5% of total protein. The main sources of vegetable protein are cereals, which make up 19.6% of total protein [33].

Colombia is simultaneously undergoing nutrition and alimentary transition [35–38]. These results make it possible to continue to characterize the alimentary and nutritional situation of the Colombian population. According to the RDA, protein intake is excessive, especially for children aged from 1 to 4 years, a time at which excess weight begins to be seen in Colombian children [39]. In the ENSIN-2015, excess protein intake is associated with excess kilocalorie intake and excess weight [39]. Nevertheless, in terms of the AMDR, intake is not excessive. For all age groups, the relative contribution of protein to total energy falls within a conservative range of 13% to 14% [5, 39]. In France, vegetable protein intake was found to be positively associated with the quality of the diet, and the quality was measured with PANDiet index [33, 40], while animal protein intake was positively or inversely associated depending on the interaction with sex and the types of foods that provided the protein source [33]. The related sociodemographic variables in the NHANES [2, 3] and the 2006-2007 INCA2 [33] are similar in terms of wealth status, but the social and economic contexts are not comparable. Here it is clear that total protein intake is positively correlated with wealth level and with other indicators of social, structural, and economic development (region and degree of urbanism). Unlike the French study, the present investigation did not determine the quality of the diet, but that reflects the particularity of each country in terms of its nutritional and dietary transitions [35–38]. Therefore, it is difficult to generalize the conclusions or dietary interventions across countries. Although total protein and its sources can have specific effects, its consumption is directly related to other components of the diet, which is a determinant of health outcomes as well as of possible effects on the environment [4]. In Colombia, iron deficiency anemia [29, 41] and vitamin B_12_ deficiency are public nutrition problems [42, 43]. The former is prevalent at early ages and is inversely correlated with age, and the latter is prevalent in adults and pregnant women. Animal proteins are essential for treating these deficiencies, and, in the case of Colombia, meat, eggs, and milk are particularly important [42–44].

### 4.1. Scope and Limitations of the Study

The primary strength of this study is that the data were taken from a nationally representative survey that provides the best estimation available to date of dietary intake in Colombia. Estimating the amounts (g) of animal and vegetable protein in preparations that are part of the FCT is complex, but the results and the consistency with what was expected when compared to other high- and medium-income countries offer external validation of the estimation process. The findings presented herein provide key elements for adjusting and designing not only dietary policies but also public policies to control more prevalent nutritional problems and to improve health in general. The main limitation of this study is the cross-sectional design of the ENSIN-2015, due to which causal relationships cannot be determined. Another limitation is the difficulty of comparing the results with other findings that have been obtained in national surveys.

## Figures and Tables

**Table 1:** Sociodemographic characteristics of the Colombian population (aged from 1 to 64 years, nonpregnant women) with estimates of the consumption of animal and vegetable protein, g/d for every 1000 kcal consumed (National Survey of Nutritional Situation in Colombia (ENSIN-2015)).

Variable	*n*	Animal protein^*∗*^		Vegetal protein^*∗*^ (plants)	
		Mean (g/d)^†^	SE	Mean (g/d) ^†^	SE
Overall	31135	19.4	0.2	13.9	0.2

Sex					
Males	15265	18.8	0.4	14.0	0.3
Females	15870	19.9	0.2	13.7	0.1

Age group (y)					
Preschool children (1–4)	6803	20.8	0.2	12.9	0.4
Schoolchildren (5–12)	6692	18.2	0.5	14.7	0.7
Adolescents (13–17)	7541	17.8	0.5	14.0	0.2
Young adults (18–26)	2147	18.6	0.3	13.6	0.2
Adults (27–49)	5332	20.2	0.4	13.7	0.2
Older adults (50–64)	2620	20.3	0.4	13.8	0.2

Height-for-age Z-score^‡^					
<−2	1995	19.6	0.6	13.8	0.2
−2 to <−1	5451	19.3	0.3	13.9	0.2
−1 to 1	11040	19.3	0.3	13.9	13.9
>1 to 2	1037	20.0	0.5	13.7	13.7
>2	252	18.7	0.6	13.6	13.6

BMI-for-age Z-score^‡^					
<−2	345	20.2	2.2	13.9	0.6
−2 to <−1	1723	19.2	0.6	14.0	0.2
−1 to 1	13004	19.3	0.2	13.9	0.2
>1 to 2	3507	19.7	0.3	13.8	0.2
>2	1177	19.8	0.5	13.7	0.3

Education of head					
<5 (primary or less)	8674	18.5	0.2	13.7	0.2
5 to <11	10630	19.6	0.4	13.9	0.2
11 to <16	9969	19.7	0.3	13.9	0.2
≥16 (university)	1661	20.3	0.6	13.7	0.2

Wealth index, quintiles^§^					
*Q*1	15631	17.6	0.3	13.7	0.5
*Q*2	7497	18.9	0.3	13.9	0.1
*Q*3	5162	20.3	0.3	14.0	0.1
*Q*4	2845	22.9	0.7	13.7	0.2

Food insecurity in the home					
No	10664	19.6	0.4	14.0	0.3
Mild	11115	19.5	0.2	13.7	0.2
Moderate	5595	18.9	0.3	13.7	0.2
Severe	3750	18.8	0.4	14.0	0.3

Urbanicity					
Big cities^||^	4454	20.4	0.7	14.6	0.5
Population from 100001 to 1000000	7411	19.5	0.3	13.8	0.1
Population from 0 to 100000	11502	19.4	0.3	13.4	0.1
Disperse population	7768	17.7	0.3	13.3	0.2

Country region					
Central	7334	18.0	0.4	14.3	0.6
Atlantic (north)	5889	18.3	0.3	12.9	0.2
Oriental	5530	20.2	0.3	13.8	0.1
Pacific (west)	4137	18.5	0.4	14.2	0.2
Bogotá	2163	21.9	1.7	14.4	0.2
Amazonia-Orinoquia	6082	20.2	0.5	13.0	0.1

^*∗*^Based on 24-hour recall. ^†^Energy-adjusted by the density method. Grams/day for every 1000 kcal consumed: 1 kcal/d = 4.18 kJ/d. ^‡^According to the WHO [25]. ^§^The wealth index is a composite measure of a household's cumulative living standard. The wealth index is calculated using easy-to-collect data on a household's ownership of selected assets such as televisions and bicycles, materials used for housing construction, type of water supply, and sanitation facilities [21]. ^||^Bogotá, Barranquilla, Medellín, Cali, and Bucaramanga.

**Table 2:** Differences in animal protein intake, g/d for every 1000 kcal consumed in Colombian population (aged from 1 to 64 years, nonpregnant women) according to sociodemographic characteristics (National Survey of Nutritional Situation in Colombia (ENSIN-2015)).

Variable	Crude difference^*∗*^ (95% CI)	*P* ^†^	Adjusted difference^‡^ (95% CI)	*P* ^§^
Sex		0.008		0.031
Males	—		—	
Females	1.1 (0.3, 1.9)		0.8 (0.1, 1.4)	
Age group (y)		0.017		0.521
Preschool children (1–4)	0.6 (−0.3, 1.5)		1.6 (0.7, 2.4)	
Schoolchildren (5–12)	−2.1 (−3.4, −0.7)		−0.8 (−1.7, 0.1)	
Adolescents (13–17)	−2.5 (−3.7, −1.3)		−1.9 (−3.0, −0.8)	
Young adults (18–26)	−1.7 (−2.8, −0.6)		−1.2 (−2.2, −0.2)	
Adults (27–49)	−0.0 (−1.2, 1.1)		0.3 (−0.8, 1.3)	
Older adults (50–64)	—		—	
Height-for-age *Z*-score^||^		0.882		0.486
<−2	0.3 (−0.7, 1.3)		0.5 (−0.4, 1.4)	
−2 to <−1	−0.1 (−0.6, 0.4)		0.1 (−0.4, 0.6)	
−1 to 1	—		—	
>1 to 2	0.7 (−0.3, 1.7)		0.5 (−0.5, 1.5)	
>2	−0.6 (−1.8, 0.7)		−0.5 (−1.6, 0.6)	
BMI-for-age *Z*-score^||^		0.594		0.871
<−2	0.9 (−3.3, 5.2)		1.0 (−3.0, 5.0)	
−2 to <−1	−0.1 (−1.0, 0.9)		0.2 (−0.8, 1.1)	
−1 to 1	—		—	
>1 to 2	0.4 (−0.2, 1.0)		0.2 (−0.3, 0.8)	
>2	0.6 (−0.4, 1.5)		0.5 (−0.4, 1.3)	
Education of head		<0.0001		0.113
<5 (primary or less)	−1.1 (−1.9, −0.2)		−0.5 (−1.2, 0.1)	
5 to <11	—		—	
11 to <16	0.1 (−0.7, 0.9)		−0.2 (−1.0, 0.7)	
≥16 (university)	0.7 (−0.6, 2.0)		0.6 (−0.8, 1.9)	
Wealth index, quintiles^¶^		<0.0001		<0.0001
*Q*1	−5.3 (−6.8, −3.8)		−4.7 (−6.0, −3.4)	
*Q*2	−4.0 (−5.5, −2.5)		−3.9 (−5.3, −2.4)	
*Q*3	−2.6 (−4.2, −1.1)		−2.6 (−4.1, −1.1)	
*Q*4	—		—	
Food insecurity		0.090		0.777
No	—		—	
Mild	−0.1 (−0.9, 0.7)		0.5 (−0.7, 0.8)	
Moderate	−0.7 (−1.6, 0.2)		−0.3 (−1.1, 0.5)	
Severe	−0.8 (−1.8, 0.2)		0.0 (−0.8, 0.9)	
Urbanicity		0.002		0.631
Big cities^*∗∗*^	—		—	
Population from 100001 to 1000000	−0.9 (−2.5, 0.7)		−0.6 (−1.4, 0.3)	
Population from 0 to 100000	−1.0 (−2.6, 0.6)		0.3 (−0.5, 1.1)	
Disperse population	−2.7 (−4.3, −1.1)		0.0 (−0.7, 0.8)	
Country region		<0.0001		0.002
Central	—		—	
Atlantic (north)	0.8 (−0.2, 1.8)		1.2 (0.4, 1.9)	
Oriental	2.2 (1.2, 3.2)		1.6 (0.9, 2.3)	
Pacific (west)	0.5 (−0.7, 1.6)		0.5 (−0.3, 1.4)	
Bogotá	3.9 (2.3, 5.6)		2.5 (1.0, 4.0)	
Amazonia-Orinoquia	2.2 (1.0, 3.4)		2.7 (1.8, 3.7)	

^*∗*^Based on 24-hour recall. Energy-adjusted by the density method. Grams/day for every 1000 kcal consumed: 1 kcal/d = 4.18 kJ/d. ^†^Test for linear trend for ordinal predictors. For sex, urbanicity, and country region, *P* is from ANOVA. All tests incorporated the complex sampling survey design. ^‡^From linear regression models with protein intake as continuous result and indicator variables in the table as predictors except for height-for-age and BMI-for-age. The estimates for education come from a model that excludes the wealth index and food security, which could be on the causal path. The wealth index estimates excluded food security. ^§^Adjusted test for linear trend or ANOVA for ordinal or categorical correlates, respectively. ^||^According to the WHO [25]. ^¶^The wealth index is a composite measure of a household's cumulative living standard. The wealth index is calculated using easy-to-collect data on a household's ownership of selected assets such as televisions and bicycles, materials used for housing construction, type of water supply, and sanitation facilities [21]. ^*∗∗*^Bogotá, Barranquilla, Medellín, Cali, and Bucaramanga.

**Table 3:** Differences in vegetal (plants) protein intake, g/d for every 1000 kcal consumed in Colombian population (aged from 1 to 64 years, nonpregnant women) according to sociodemographic characteristics (National Survey of Nutritional Situation in Colombia (ENSIN-2015)).

Variable	Crude difference^*∗*^ (95% CI)	*P* ^†^	Adjusted difference^‡^ (95% CI)	*P* ^§^
Sex		0.518		0.443
Males	—		—	
Females	−0.2 (−1.0, 0.5)		−0.2 (−0.7, 0.3)	
Age group (y)		0.621		0.950
Preschool children (1–4)	−1.0 (−1.9, −0.1)		−1.1 (−2.1, −0.1)	
Schoolchildren (5–12)	0.9 (−0.4, 2.3)		0.6 (−0.1, 1.3)	
Adolescents (13–17)	0.2 (−0.3, 0.7)		0.1 (−0.4, 0.5)	
Young adults (18–26)	−0.3 (−0.7, 0.2)		−0.3 (−0.8, 0.1)	
Adults (27–49)	−0.1 (−0.7, 0.4)		−0.2 (−0.7, 0.3)	
Older adults (50–64)	—		—	
Height-for-age *Z*-score^||^		0.950		0.576
<−2	−0.1 (−0.5, 0.3)		−0.0 (−0.4, 0.3)	
−2 to <−1	−0.1 (−0.3, 0.1)		−0.0 (−0.3, 0.2)	
−1 to 1	—		—	
>1 to 2	−0.3 (−0.6, 0.1)		−0.3 (−0.7, 0.1)	
>2	−0.4 (−1.0, 0.3)		−0.4 (−1.1, 0.3)	
BMI-for-age *Z*-score^||^		0.349		0.265
<−2	−0.0 (−1.1, 1.1)		−0.1 (−1.2, 1.1)	
−2 to <−1	0.1 (−0.2, 0.4)		1.1 (−0.2, 0.4)	
−1 to 1	—		—	
>1 to 2	−0.1 (−0.4, 0.2)		−0.1 (−0.4, 0.1)	
>2	−0.2 (−0.6, 0.3)		−0.2 (−0.6, 0.1)	
Education of head		0.363		0.254
<5 (primary or less)	−0.2 (−0.6, 0.1)		−0.0 (−0.3, 0.3)	
5 to <11	—		—	
11 to <16	0.0 (−0.3, 0.3)		−0.1 (−0.4, 0.1)	
≥16 (university)	−0.2 (−0.7, 0.2)		−0.3 (−0.7, 0.2)	
Wealth index, quintiles^¶^		0.894		0.210
*Q*1	0.0 (−1.0, 1.1)		0.9 (−0.4, 2.2)	
*Q*2	0.2 (−0.2, 0.7)		0.5 (0.0, 1.0)	
*Q*3	0.3 (−0.2, 0.8)		0.4 (−0.1, 0.9)	
*Q*4	—		—	
Food insecurity		0.661		0.930
No	—		—	
Mild	−0.3 (−0.6, −0.0)		−0.3 (−0.5, −0.0)	
Moderate	−0.3 (−0.7, 0.1)		−0.2 (−0.6, 0.1)	
Severe	0.0 (−0.5, 0.6)		0.1 (−0.4, 0.7)	
Urbanicity		0.009		0.026
Big cities^*∗∗*^	—		—	
Population from 100001 to 1000000	−0.8 (−1.8, 0.1)		−0.9 (−1.9, 0.2)	
Population from 0 to 100000	−1.3 (−2.2, −0.3)		−1.5 (−2.6, −0.3)	
Disperse population	−1.3 (−2.3, −0.3)		−1.7 (−3.3, −0.2)	
Country region		0.740		0.941
Central	—		—	
Atlantic (north)	−1.4 (−2.7, −0.1)		−1.4 (−2.3, −0.5)	
Oriental	−0.6 (−1.8, 0.6)		−0.2 (−0.7, 0.4)	
Pacific (west)	−0.1 (−1.4, 1.1)		−0.1 (−0.8, 0.7)	
Bogotá	0.0 (−1.2, 1.3)		−0.5 (−1.5, 0.5)	
Amazonia-Orinoquia	−1.3 (−2.6, −0.1)		−1.0 (−1.6, −0.4)	

^*∗*^Based on 24-hour recall. Energy-adjusted by the density method. Grams/day for every 1000 kcal consumed: 1 kcal/d = 4.18 kJ/d. ^†^Test for linear trend for ordinal predictors. For sex, urbanicity, and country region, *P* is from ANOVA. All tests incorporated the complex sampling survey design. ^‡^From linear regression models with protein intake as continuous result and indicator variables in the table as predictors except for height-for-age and BMI-for-age. The estimates for education come from a model that excludes the wealth index and food security, which could be on the causal path. The wealth index estimates excluded food security. ^§^Adjusted test for linear trend or ANOVA for ordinal or categorical correlates, respectively. ^||^According to the WHO [25]. ^¶^The wealth index is a composite measure of a household's cumulative living standard. The wealth index is calculated using easy-to-collect data on a household's ownership of selected assets such as televisions and bicycles, materials used for housing construction, type of water supply, and sanitation facilities [21]. ^*∗∗*^Bogotá, Barranquilla, Medellín, Cali, and Bucaramanga.

**Table 4:** Relative contribution (%)^*∗*^ of the main sources to the total animal and vegetable protein in the Colombian population (aged from 1 to 64 years) (National Survey of Nutritional Situation in Colombia (ENSIN-2015)).

Food	%				
	All (31135)	1 to 4 y (6803)	5 to 17 y (14233)	18 to 64 y (10099)	Pregnant women (1322)
*Animal*					
Beef	17.8	12.1	18.6	19.4	13.4
Chicken meat (poultry)	16.3	15.1	16.0	17.0	10.8
Eggs	10.5	13.0	11.7	9.3	8.5
Fish and shellfish	9.1	8.3	9.1	9.5	11.5
Whole milk	7.1	15.8	7.7	5.3	5.6
Cheese	5.5	4.9	6.5	5.4	4.9
Pork meat	5.2	2.8	5.1	6.4	4.3
Organ meats and other cuts of beef, pork (viscera)	4.5	4.0	4.6	4.9	7.4
Noncanned processed meats	4.1	3.5	5.1	3.8	6.3
Cereals^†^	3.4	2.4	2.8	3.9	6.0
Bread, arepa, pasta^†^	3.3	2.2	3.0	3.7	4.5
Potatoes^†^	1.4	1.5	0.8	1.7	2.2
Legumes and derived products^†^	1.0	1.3	1.0	0.9	2.1
Cereal-based preparations, roots^†^	0.9	0.7	1.2	1.0	0.7
Banana preparations^†^	0.9	1.4	0.9	0.9	1.7
Fermented milks	0.8	2.1	0.9	0.4	0.8

*Vegetal*					
Bread, arepa, pasta	20.0	17.3	21.9	20.5	16.8
Cereals	19.8	19.9	22.0	19.5	16.2
Legumes and derived products	8.2	7.1	9.1	7.7	6.3
Potatoes	6.7	8.0	6.5	6.9	5.2
Vegetable	4.8	4.9	3.8	5.4	5.8
Noncanned processed meats^‡^	4.8	4.5	5.8	4.5	5.4
Derivatives of industrialized cereals	4.5	6.1	4.7	3.4	4.6
Fruit	4.1	5.7	3.1	4.2	6.4
Cereal-based preparations, roots	2.9	1.4	3.5	3.1	2.6
Banana preparations	2.7	3.0	2.8	2.8	3.6
Home-made drinks with water and milk (coffee)^‡^	1.9	1.1	1.7	2.4	1.4
Packaged foods (industrialized)	1.2	1.1	1.9	0.6	0.6
Home-made and industrialized desserts^‡^	1.2	1.2	1.3	0.9	1.5
Natural fruit juice	1.1	0.6	1.1	1.2	1.1
Roots, tubers	0.9	0.8	0.7	1.2	1.4
Supplements and add-ons	0.5	0.8	0.3	0.5	2.1

(*n*). ^*∗*^For animal protein: (total animal protein per item/total animal protein) × 100. For vegetal protein: (total vegetable protein per item/total vegetable protein) × 100. ^†^Only the animal component in the preparation. ^‡^Only the vegetal component in the preparation.

## Data Availability

To access the ENSIN 2015 public database, we must register in the repository of the Ministry of Public Health, repositorio@minsalud.gov.co, and make the request through the format available at https://www.minsalud.gov.co/sites/rid/Listas/BibliotecaDigital/RIDE/VS/ED/GCFI/forma-ensin-2015.zip. The databases used to translate food consumption into proteins of animal or vegetable origin can be requested from the authors.

## Supplementary Materials

Supplementary Materials There are 18 tables in the supplementary file. In the first five tables 1S to 5S, the details of the total consumption of animal and vegetable proteins are presented by sex and age groups (1–64 y, 1–4 y, 5–12 y, 13–17 y, and 18–64 y). The first five tables contain the following: g/d, g per 1000 kcal, the relative contribution to energy of protein consumption (%), and the relative contribution of animal and vegetable protein consumption to total protein (%), in addition to the protein consumption per kg of current and theoretical ideal weight. Table 6S shows the relative contribution (%) of the main food sources to total protein in the Colombian population by age groups. Tables 7S to 18S present in detail the mean consumption of proteins of animal and vegetable origin for each of the categories of the covariates studied and the crude and adjusted differences in these covariates. Tables 7S to 18S present the information for each age group of interest (1–4 y, 5–12 y, 13–17 y, and 18–64 y).

## References

[1] Institute of Medicine (2005). *Dietary Reference Intakes for Energy, Carbohydrate, Fiber, Fat, Fatty Acids, Cholesterol, Protein, and Amino Acids (Macronutrients)*.

[2] Shan Z., Rehm C. D., Rogers G. (2019). Trends in dietary carbohydrate, protein, and fat intake and diet quality among US adults, 1999–2016. *JAMA*.

[3] Pasiakos S., Agarwal S., Lieberman H., Fulgoni V. (2015). Sources and amounts of animal, dairy, and plant protein intake of US adults in 2007–2010. *Nutrients*.

[4] Willett W., Rockström J., Loken B. (2019). Food in the anthropocene: the EAT-lancet commission on healthy diets from sustainable food systems. *The Lancet*.

[5] Wolfe R. R., Cifelli A. M., Kostas G., Kim I.-Y. (2017). Optimizing protein intake in adults: interpretation and application of the recommended dietary allowance compared with the acceptable macronutrient distribution range. *Advances in Nutrition: An International Review Journal*.

[6] Wu G. (2016). Dietary protein intake and human health. *Food & Function*.

[7] Song M., Fung T. T., Hu F. B. (2016). Association of animal and plant protein intake with all-cause and cause-specific mortality. *JAMA Internal Medicine*.

[8] Naghshi S., Sadeghi O., Willett W. C., Esmaillzadeh A. (2020). Dietary intake of total, animal, and plant proteins and risk of all cause, cardiovascular, and cancer mortality: systematic review and dose-response meta-analysis of prospective cohort studies. *BMJ*.

[9] Abete I., Romaguera D., Vieira A. R., Lopez De Munain A., Norat T. (2014). Association between total, processed, red and white meat consumption and all-cause, CVD and IHD mortality: a meta-analysis of cohort studies. *British Journal of Nutrition*.

[10] Chen G.-C., Lv D.-B., Pang Z., Liu Q.-F. (2013). Red and processed meat consumption and risk of stroke: a meta-analysis of prospective cohort studies. *European Journal of Clinical Nutrition*.

[11] Shang X., Scott D., Hodge A. (2017). Dietary protein from different food sources, incident metabolic syndrome and changes in its components: an 11-year longitudinal study in healthy community-dwelling adults. *Clinical Nutrition*.

[12] Minsalud ICBF (2021). Metodología ENSIN-2015. https://www.minsalud.gov.co/sites/rid/Lists/BibliotecaDigital/RIDE/VS/ED/GCFI/documento-metodologico-ensin-2015.pdf.

[13] Moshfegh A. J., Rhodes D. G., Baer D. J. (2008). The US department of agriculture automated multiple-pass method reduces bias in the collection of energy intakes. *The American Journal of Clinical Nutrition*.

[14] Blanton C. A., Moshfegh A. J., Baer D. J., Kretsch M. J. (2006). The USDA automated multiple-pass method accurately estimates group total energy and nutrient intake. *Journal of Nutrition*.

[15] Gaurth Hansen R., Wyse B. W. (1980). Expression of nutrient allowances per 1,000 kilocalories. *Journal of the American Dietetic Association*.

[16] Vernarelli J. A., Mitchell D. C., Rolls B. J., Hartman T. J. (2013). Methods for calculating dietary energy density in a nationally representative sample. *Procedia Food Science*.

[17] (2020). AMPM—USDA automated multiple-pass method: USDA ARS. https://www.ars.usda.gov/northeast-area/beltsville-md-bhnrc/beltsville-human-nutrition-research-center/food-surveys-research-group/docs/ampm-usda-automated-multiple-pass-method/.

[18] Conway J. M., Ingwersen L. A., Vinyard B. T., Moshfegh A. J. (2003). Effectiveness of the US department of agriculture 5-step multiple-pass method in assessing food intake in obese and nonobese women. *The American Journal of Clinical Nutrition*.

[19] Lauritsen J. (1998). *Diet Cancer and, Society) Health Project*.

[20] FAO (2018). *Escala Latinoamericana y Caribeña de Seguridad Alimentaria (ELCSA)—Manual de uso y Aplicación*.

[21] Rutstein S. O. (2008). The DHS wealth index: approaches for rural and urban areas. *Demographic and Health Research*.

[22] Programa de las Naciones Unidas Para el Desarrollo (PNUD) (2013). *Informe de Desarrollo Humano*.

[23] DPN (2014). *Plan Nacional de Desarrollo, 2014–2018*.

[24] CDC (2011). *Measuring Children’s Height and Weight Accurately at Home*.

[25] WHO—World Health Organization (2011). *Patrones de Crecimiento Infantil de la OMS*.

[26] Taylor C. L., Carriquiry A. L., Bailey R. L., Sempos C. T., Yetley E. A. (2013). Appropriateness of the probability approach with a nutrient status biomarker to assess population inadequacy: a study using vitamin D. *The American Journal of Clinical Nutrition*.

[27] StataCorp (2015). *Stata Statistical Software: Release 14*.

[28] World Medical Association (2015). Declaración de helsinki de la AMM—ethical principles for human medical research. https://www.wma.net/es/policies-post/declaracion-de-helsinki-de-la-amm-principios-eticos-para-las-investigaciones-medicas-en-seres-humanos/.

[29] (2019). Encuesta nacional de la situación nutricional-ensin 2015. https://www.icbf.gov.co/bienestar/nutricion/encuesta-nacional-situacion-nutricional#ensin3.

[30] Profamilia (2005). *Encuesta Nacional de la Situación Nutricional de Colombia*.

[31] Herrán O., DelCastillo S., Patiño G. A. (2017). Exceso de proteínas en la pobreza: la paradoja del exceso de peso en niños colombianos. *Revista Chilena de Nutricion*.

[32] Robinson S. L., Mora-Plazas M., Oliveros H., Marin C., Lozoff B., Villamor E. (2021). Dietary patterns in middle childhood and behavior problems in adolescence. *European Journal of Clinical Nutrition*.

[33] Camilleri G. M., Verger E. O., Huneau J.-F., Carpentier F., Dubuisson C., Mariotti F. (2013). Plant and animal protein intakes are differently associated with nutrient adequacy of the diet of French adults. *Journal of Nutrition*.

[34] (2021). FAOSTAT (2021). http://www.fao.org/faostat/es/#home.

[35] Kasper N. M., Herrán O. F., Villamor E. (2014). Obesity prevalence in Colombian adults is increasing fastest in lower socio-economic status groups and urban residents: results from two nationally representative surveys. *Public Health Nutrition*.

[36] Herrán O. F., Villamor E., Quintero-Lesmes D. C. (2019). Adherence to a snacking dietary pattern is decreasing in Colombia among the youngest and the wealthiest: results of two representative national surveys. *BMC Public Health*.

[37] Quintero-Lesmes D. C., Herran O. F. (2019). Food changes and geography: dietary transition in Colombia. *Annals of Global Health*.

[38] Herrán Ó. F., Patiño G. A., Del Castillo S. E. (2010). La transición alimentaria y el exceso de peso en adultos evaluados con base en la encuesta de la situación nutricional en Colombia. *Biomedica*.

[39] Herrán O. F., Gamboa-Delgado E. M. (2020). Trends of adherence to dietary patterns in Colombian population 2010–2015. *American Journal of Health Behavior*.

[40] Verger E. O., Mariotti F., Holmes B. A., Paineau D., Huneau J.-F. (2012). Evaluation of a diet quality index based on the probability of adequate nutrient intake (PANDiet) using national French and US dietary surveys. *PLoS One*.

[41] Ramírez-Vélez R., Correa-Bautista J., Martínez-Torres J., González-Ruíz K., Lobelo F. (2016). Ferritin levels in Colombian children: findings from the 2010 national nutrition survey (ENSIN). *International Journal of Environmental Research and Public Health*.

[42] Villamor E., Mora-Plazas M., Forero Y., Lopez-Arana S., Baylin A. (2008). Vitamin B-12 status is associated with socioeconomic level and adherence to an animal food dietary pattern in Colombian school children. *Journal of Nutrition*.

[43] Herrán O. F., Ward J. B., Villamor E. (2015). Vitamin B12 serostatus in Colombian children and adult women: results from a nationally representative survey. *Public Health Nutrition*.

[44] Herrán O. F., Bermúdez J. N., Del Pilar Zea M. (2020). Red meat and egg intake and serum ferritin concentrations in Colombian children: results of a population survey, ENSIN-2015. *Journal of nutritional science*.

