# Supplementary material for: Intake of Animal Protein and Dietary Sources in the Colombian Population: Results of the National Nutrition Survey (ENSIN-2015)
Source: J Nutr Metab. 2022 Jan 13;2022:2345400. doi: 10.1155/2022/2345400 (PMC8776471; doi:10.1155/2022/2345400)
Supplement: Supplementary Materials — There are 18 tables in the supplementary file. In the first five tables 1S to 5S, the details of the total consumption of animal and vegetable proteins are presented by sex and age groups (1–64 y, 1–4 y, 5–12 y, 13–17 y, and 18–64 y). The first five tables contain the following: g/d, g per 1000 kcal, the relative contribution to energy of protein consumption (%), and the relative contribution of animal and vegetable protein consumption to total protein (%), in addition to the protein consumption per kg of current and theoretical ideal weight. Table 6S shows the relative contribution (%) of the main food sources to total protein in the Colombian population by age groups. Tables 7S to 18S present in detail the mean consumption of proteins of animal and vegetable origin for each of the categories of the covariates studied and the crude and adjusted differences in these covariates. Tables 7S to 18S present the information for each age group of interest (1–4 y, 5–12 y, 13–17 y, and 18–64 y). [file 2345400.f1.docx]

**Intake of animal protein and dietary sources in the colombian population:**

**results of the National Nutrition Survey. ENSIN, 2015**

Oscar F. Herrán^1^, María del Pila Zea^2^

^1^ Escuela de Nutrición y Dietética, Universidad Industrial de Santander, Carrera 32 No. 29-31, Bucaramanga, Santander, Colombia. [herran@uis.edu.co] ^2^ Universidad Javeriana de Cali. Facultad de Ciencias de la Salud. Calle 17 # 121B-80, Cali, Valle del Cauca, Colombia..

Online Supplementary Material

|  | Page |
| --- | --- |
| **Table 1S** Total energy, animal, and vegetal protein intakes in the Colombian population (*1-64 y, non-pregnant women*). National Survey of Nutritional Situation in Colombia (ENSIN, 2015). | 4 |
| **Table 2S** Total energy, animal, and vegetal protein intakes in the Colombian population (*1-4 y, non-pregnant women*). National Survey of Nutritional Situation in Colombia (ENSIN, 2015). | 5 |
| **Table 3S** Total energy, animal, and vegetal protein intakes in the Colombian population (*5-12 y, non-pregnant women*). National Survey of Nutritional Situation in Colombia (ENSIN, 2015). | 6 |
| **Table 4S** Total energy, animal, and vegetal protein intakes in the Colombian population (*13-17 y, non-pregnant women*). National Survey of Nutritional Situation in Colombia (ENSIN, 2015). | 7 |
| **Table 5S** Total energy, animal, and vegetal protein intakes in the Colombian population (*18-64 y, non-pregnant women*). National Survey of Nutritional Situation in Colombia (ENSIN, 2015). | 8 |
| **Table 6S** Relative contribution (%)* of the main sources to the total protein in Colombian population (*1 to 64 y*). National Survey of Nutritional Situation in Colombia (ENSIN, 2015). | 9 |
| **Table 7S** Sociodemographic characteristics of the Colombian population (*1* to *4* years, non-pregnant women) with estimates of the consumption of animal and vegetable protein, g/d for every 1000 kcal consumed, in the National Survey of Nutritional Situation in Colombia (ENSIN, 2015). | 10 |
| **Table 8S** Differences in animal protein intake, g/d for every 1000 kcal consumed, in Colombian population (*1 to 4 years, non-pregnant women*) according to sociodemographic characteristics. National Survey of Nutritional Situation in Colombia (ENSIN, 2015). | 11 |
| **Table 9S** Differences in vegetal (Plants) protein intake, g/d for every 1000 kcal consumed, in Colombian population (*1 to 4 years, non-pregnant women*) according to sociodemographic characteristics. National Survey of Nutritional Situation in Colombia (ENSIN, 2015). | 12 |
| **Table 10S** Sociodemographic characteristics of the Colombian population (*5 to 12 years, non-pregnant women*) with estimates of the consumption of animal and vegetable protein, g/d for every 1000 kcal consumed, in the National Survey of Nutritional Situation in Colombia (ENSIN, 2015). | 13 |
| **Table 11S** Differences in animal protein intake, g/d for every 1000 kcal consumed, in Colombian population (5 *to 12 years, non-pregnant women*) according to sociodemographic characteristics. National Survey of Nutritional Situation in Colombia (ENSIN, 2015). | 14 |
| **Table 12S** Differences in vegetal (Plants) protein intake, g/d for every 1000 kcal consumed, in Colombian population (*5 to 12 years, non-pregnant women*) according to sociodemographic characteristics. National Survey of Nutritional Situation in Colombia (ENSIN, 2015). | 15 |
| **Table 13S** Sociodemographic characteristics of the Colombian population (*13 to 17 years, non-pregnant women*) with estimates of the consumption of animal and vegetable protein, g/d for every 1000 kcal consumed, in the National Survey of Nutritional Situation in Colombia (ENSIN, 2015). | 16 |
| **Table 14S** Differences in animal protein intake, g/d for every 1000 kcal consumed, in Colombian population (*13 to 17 years, non-pregnant women*) according to sociodemographic characteristics. National Survey of Nutritional Situation in Colombia (ENSIN, 2015). | 17 |
| **Table 15S** Differences in vegetal (Plants) protein intake, g/d for every 1000 kcal consumed, in Colombian population (*13 to 17 years, non-pregnant women*) according to sociodemographic characteristics. National Survey of Nutritional Situation in Colombia (ENSIN, 2015). | 18 |
| **Table 16S** Sociodemographic characteristics of the Colombian population (*18 to 64 years, non-pregnant women*) with estimates of the consumption of animal and vegetable protein, g/d for every 1000 kcal consumed, in the National Survey of Nutritional Situation in Colombia (ENSIN, 2015). | 19 |
| **Table 17S** Differences in animal protein intake, g/d for every 1000 kcal consumed, in Colombian population (*18 to 64 years, non-pregnant women*) according to sociodemographic characteristics. National Survey of Nutritional Situation in Colombia (ENSIN, 2015). | 20 |
| **Table 18S** Differences in vegetal (Plants) protein intake, g/d for every 1000 kcal consumed, in Colombian population (*18 to 64 years, non-pregnant women*) according to sociodemographic characteristics. National Survey of Nutritional Situation in Colombia (ENSIN, 2015). | 21 |

| **Table 1S** Total energy, animal, and vegetal protein intakes in the Colombian population (*1-64 y, non-pregnant women*). National Survey of Nutritional Situation in Colombia (ENSIN, 2015). | | | | |
| --- | --- | --- | --- | --- |
|  | All  [n = 31135] | Men  [n = 15265] | Women  [n = 15870] | *P*† |
| Actual intake | Mean (95% CI)* | Mean (95% CI)* | Mean (95% CI)* |  |
| Total energy, kcal/d‡ | 1962 (1906, 2018) | 2083 (1977 2200) | 1843 (1787, 1900) | <0.0001 |
| Total protein, g/d | 63.7 (61.7, 65.6) | 67.0 (63.0, 71.0) | 60.4 (58.4, 62.3) | 0.003 |
| Total protein-1000, g/d§ | 32.9 (32.4, 33.4) | 32.6 (31.9, 33.3) | 33.1 (32.7, 33.6) | 0.205 |
| Total protein, % of energy | 13.2 (13.0, 13.3) | 13.1 (12.8, 13.3) | 13.3 (13.1, 13.5) | 0.205 |
| Total protein / kg current weight, g/d | 1.53 (1.43, 1.63) | 1.56 (1.47, 1.65) | 1.50 (1.33, 1.67) | 0.550 |
| Total protein / kg ideal theoretical weight, g/d | 1.65 (1.54, 1.76) | 1.69 (1.56, 1.81) | 1.61 (1.45, 1.78) | 0.492 |
|  |  |  |  |  |
| Animal protein, g/d | 36.9 (35.4, 38.3) | 38.0 (35.2, 40.9) | 35.8 (34.6, 36.9) | 0.144 |
| Animal protein, % of energy | 7.75 (7.56, 7.95) | 7.52 (7.22, 7.82) | 7.97 (7.82, 8.12) | 0.008 |
| Animal protein-1000, g/d§ | 19.4 (18.4, 19.4) | 18.8 (18.0, 19.6) | 19.9 (19.5, 20.3) | 0.008 |
| Animal protein, % of total protein | 56.6 (55.1, 58.0) | 53.1 (50.3, 55.9) | 60.0 (58.3, 61.7) | <0.0001 |
| Animal protein-1000 / kg current weight, g/d | 0.49 (0.47, 0.52) | 0.48 (0.44, 0.51) | 0.51 (0.47, 0.55) | 0.266 |
| Animal protein-1000 / kg ideal theoretical weight, g/d | 0.53 (0.50, 0.56) | 0.52 (0.47, 0.57) | 0.55 (0.51, 0.59) | 0.318 |
|  |  |  |  |  |
| Vegetal protein, g/d | 26.9 (26.3, 27.4) | 28.7 (27.9, 29.6) | 25.1 (24.3, 25.8) | <0.0001 |
| Vegetal protein, % of energy | 5.54 (5.39, 5.69) | 5.59 (5.31, 5.87) | 5.50 (5.40, 5.59) | 0.518 |
| Vegetal protein-1000, g/d§ | 13.9 (13.5, 14.2) | 14.0 (13.3, 14.7) | 13.7 (13.5, 14.0) | 0.518 |
| Vegetal protein, % of total protein | 43.8 (42.7, 44.9) | 44.6 (42.5, 46.6) | 43.1 (42.3, 43.8) | 0.167 |
| Vegetal protein-1000 / kg current weight, g/d | 0.35 (0.32, 0.39) | 0.36 (0.30, 0.42) | 0.35 (0.33, 0.37) | 0.819 |
| Vegetal protein-1000 / kg ideal theoretical weight, g/d | 0.38 (0.34, 0.43) | 0.39 (0.31, 0.47) | 0.38 (0.36, 0.40) | 0.795 |
| [n]  *Based on a 24-Hour Recall: Values are means and 95% CI, weighted for the survey design.  †*P* values for differences between men and women, as assessed by using Student´s t test.  ‡1 kcal/d = 4.18 kJ/d  §Energy-Adjusted by the density method [g / d per 1000 kcal] | | | | |

| **Table 2S** Total energy, animal, and vegetal protein intakes in the Colombian population (*1-4 y, non-pregnant women*). National Survey of Nutritional Situation in Colombia (ENSIN, 2015). | | | | |
| --- | --- | --- | --- | --- |
|  | All  [n = 6923] | Men  [n =3188] | Women  [n =3735] | *P*† |
|  | Mean (95% CI)* | Mean (95% CI)* | Mean (95% CI)* |  |
| Total energy, kcal/d‡ | 1677 (1449, 1905) | 1569 (1528, 1610) | 1755 (1400, 2111) | 0.301 |
| Total protein, g/d | 54.5 (48.4, 60.6) | 52.2 (50.7, 53.7) | 56.1 (46.4, 65.8) | 0.424 |
| Total protein-1000, g/d§ | 32.9 (32.1, 33.7) | 33.5 (33.0, 34.0) | 32.4 (31.3, 33.6) | 0.082 |
| Total protein, % of energy | 13.2 (12.8, 13.5) | 13.4 (13.2, 13.6) | 13.0 (12.5, 13.4) | 0.082 |
| Total protein / kg current weight, g/d | 3.95 (3.68, 4.22) | 3.84 (3.71, 3.96) | 4.03 (3.60, 4.46) | 0.389 |
| Total protein / kg ideal theoretical weight, g/d | 4.06 (3.81, 4.30) | 3.96 (3.83, 4.09) | 4.13 (3.75, 4.50) | 0.422 |
|  |  |  |  |  |
| Animal protein, g/d | 34.2 (30.0, 38.4) | 32.7 (31.7, 33.7) | 35.3 (28.6, 42.0) | 0.444 |
| Animal protein, % of energy | 8.33 (8.14, 8.51) | 8.50 (8.30, 8.69) | 8.20 (7.97, 8.43) | 0.051 |
| Animal protein-1000, g/d§ | 20.8 (20.4, 21.3) | 21.2 (20.8, 21.7) | 20.5 (19.9, 21.1) | 0.051 |
| Animal protein, % of total protein | 62.6 (61.7, 63.6) | 62.5 (61.8, 63.2) | 62.7 (61.2, 64.2) | 0.776 |
| Animal protein-1000 / kg current weight, g/d | 1.55 (1.46, 1.64) | 1.59 (1.54, 1.65) | 1.52 (1.39, 1.65) | 0.269 |
| Animal protein-1000 / kg ideal theoretical weight, g/d | 1.60 (1.50, 1.70) | 1.64 (1.59, 1.70) | 1.56 (1.41, 1.71) | 0.289 |
|  |  |  |  |  |
| Vegetal protein, g/d | 21.1 (19.7, 22.5) | 20.5 (20.0, 21.1) | 21.5 (19.2, 23.7) | 0.417 |
| Vegetal protein, % of energy | 5.14 (4.82, 5.46) | 5.28 (5.18, 5.37) | 5.04 (4.54, 5.55) | 0.371 |
| Vegetal protein-1000, g/d§ | 12.9 (12.1, 13.6) | 13.2 (13.0, 13.4) | 12.6 (11.3, 13.9) | 0.371 |
| Vegetal protein, % of total protein | 40.0 (38.1, 42.0) | 40.7 (39.8, 41.5) | 39.6 (36.4, 42.7) | 0.494 |
| Vegetal protein-1000 / kg current weight, g/d | 0.96 (0.87, 1.04) | 0.98 (0.96, 1.00) | 0.94 (0.80, 1.07) | 0.537 |
| Vegetal protein-1000 / kg ideal theoretical weight, g/d | 0.98 (0.89, 1.08) | 1.01 (0.99, 1.03) | 0.96 (0.81, 1.11) | 0.514 |
| [n]  *Based on a 24-Hour Recall: Values are means and 95% CI, weighted for the survey design.  †*P* values for differences between men and women, as assessed by using Student´s t test.  ‡1 kcal/d = 4.18 kJ/d  §Energy-Adjusted by the density method [g / d per 1000 kcal] | | | | |

| **Table 3S** Total energy, animal, and vegetal protein intakes in the Colombian population (*5-12 y, non-pregnant women*). National Survey of Nutritional Situation in Colombia (ENSIN, 2015). | | | | |
| --- | --- | --- | --- | --- |
|  | All  [n = 6692] | Men  [n =3782] | Women  [n =2910] | *P*† |
|  | Mean (95% CI)* | Mean (95% CI)* | Mean (95% CI)* |  |
| Total energy, kcal/d‡ | 1814 (1646, 1982) | 1829 (1555, 2103) | 1789 (17.5, 1873) | 0.780 |
| Total protein, g/d | 58.0 (52.1, 63.9) | 59.0 (49.1, 68.8) | 56.5 (53.4, 59.5) | 0.632 |
| Total protein-1000, g/d§ | 32.1 (31.5, 32.7) | 32.3 (31.4, 33.1) | 31.8 (31.1, 32.5) | 0.361 |
| Total protein, % of energy | 12.8 (12.6, 13.1) | 12.9 (12.6, 13.3) | 12.7 (12.4, 13.0) | 0.361 |
| Total protein / kg current weight, g/d | 2.15 (2.01, 2.29) | 2.17 (1.94, 2.41) | 2.12 (2.02, 2.22) | 0.662 |
| Total protein / kg ideal theoretical weight, g/d | 2.32 (2.25, 2.39) | 2.39 (2.27, 2.51) | 2.20 (2.10, 2.30) | 0.015 |
|  |  |  |  |  |
| Animal protein, g/d | 32.9 (28.2, 37.6) | 32.6 (25.4, 39.8) | 33.4 (31.6, 35.1) | 0.837 |
| Animal protein, % of energy | 7.27 (6.85, 7.70) | 7.11 (6.56, 7.67) | 7.54 (7.28, 7.82) | 0.159 |
| Animal protein-1000, g/d§ | 18.2 (17.1, 19.3) | 17.9 (16.4, 19.2) | 18.9 (18.2, 19.6) | 0.159 |
| Animal protein, % of total protein | 55.8 (53.2, 58.4) | 54.3 (51.3, 57.4) | 58.3 (57.2, 59.4) | 0.017 |
| Animal protein-1000 / kg current weight, g/d | 0.68 (0.66, 0.71) | 0.67 (0.64, 0.70) | 0.71 (0.68, 0.74) | 0.058 |
| Animal protein-1000 / kg ideal theoretical weight, g/d | 0.74 (0.72, 0.77) | 0.74 (0.71, 0.78) | 0.74 (0.71, 0.78) | 0.893 |
|  |  |  |  |  |
| Vegetal protein, g/d | 26.2 (25.2, 27.3) | 27.2 (25.6, 28.8) | 24.7 (23.6, 25.8) | 0.012 |
| Vegetal protein, % of energy | 5.89 (5.38, 6.42) | 6.09 (5.40, 6.78) | 5.59 (5.38, 5.80) | 0.172 |
| Vegetal protein-1000, g/d§ | 14,7 (13.4, 16.1) | 15,2 (13.5, 17.0) | 14.0 (13.4, 14.5) | 0.172 |
| Vegetal protein, % of total protein | 47.1 (43.5, 50.7) | 48.2 (43.3, 53.1) | 45.3 (43.1, 47.4) | 0.281 |
| Vegetal protein-1000 / kg current weight, g/d | 0.57 (0.51, 0.63) | 0.58 (0.50, 0.66) | 0.55 (0.48, 0.62) | 0.584 |
| Vegetal protein-1000 / kg ideal theoretical weight, g/d | 0.62 (0.52, 0.72) | 0.65 (0.51, 0.79) | 0.57 (0.51, 0.64) | 0.335 |
| [n]  *Based on a 24-Hour Recall: Values are means and 95% CI, weighted for the survey design.  †*P* values for differences between men and women, as assessed by using Student´s t test.  ‡1 kcal/d = 4.18 kJ/d  §Energy-Adjusted by the density method [g / d per 1000 kcal] | | | | |

| **Table 4S** Total energy, animal, and vegetal protein intakes in the Colombian population (*13-17 y, non-pregnant women*). National Survey of Nutritional Situation in Colombia (ENSIN, 2015). | | | | |
| --- | --- | --- | --- | --- |
|  | All  [n = 7541] | Men  [n = 3545] | Women  [n = 3996] | *P*† |
|  | Mean (95% CI)* | Mean (95% CI)* | Mean (95% CI)* |  |
| Total energy, kcal/d‡ | 2260 (2156, 2364) | 2374 (2302, 2447) | 2158 (1962, 2354) | 0.043 |
| Total protein, g/d | 71.4 (66.0, 76.8) | 73.3 (71.1, 75,6) | 69.7 (59.2, 80.1) | 0.497 |
| Total protein-1000, g/d§ | 31.8 (30.6, 33.0) | 31.3 (30.6, 32.0) | 32.3 (30.2, 34.4) | 0.370 |
| Total protein, % of energy | 12.7 (12.2, 13.2) | 12.5 (12.3, 12.8) | 12.9 (12.1, 13.8) | 0.370 |
| Total protein / kg current weight, g/d | 1.41 (1.25, 1.57) | 1.38 (1.34, 1.43) | 1.44 (1.14, 1.73) | 0.723 |
| Total protein / kg ideal theoretical weight, g/d | 1.44 (1.29, 1.60) | 1.42 (1.38, 1.46) | 1.47 (1.18, 1.75) | 0.752 |
|  |  |  |  |  |
| Animal protein, g/d | 39.2 (35.1, 42.5) | 39.3 (37.5, 41.1) | 39.1 (32.9, 45.3) | 0.944 |
| Animal protein, % of energy | 7.10 (6.73, 7.47) | 6.82 (6.50, 7.14) | 7.38 (6.79, 7.92) | 0.103 |
| Animal protein-1000, g/d§ | 17.8 (16.8, 18.7) | 17.0 (16.2, 17.8) | 18.4 (17.0, 19.8) | 0.103 |
| Animal protein, % of total protein | 54.6 (53.0, 56.1) | 53.2 (51.6, 54.9) | 55.7 (53.5, 58.0) | 0.083 |
| Animal protein-1000 / kg current weight, g/d | 0.35 (0.32, 0.38) | 0.32 (0.31, 0.33) | 0.37 (0.33, 0.43) | 0.029 |
| Animal protein-1000 / kg ideal theoretical weight, g/d | 0.36 (0.33, 0.39) | 0.33 (0.32, 0.34) | 0.38 (0.34, 0.43) | 0.024 |
|  |  |  |  |  |
| Vegetal protein, g/d | 31.6 (29.6, 33.5) | 33.2 (31.8, 34.5) | 30.1 (26.4, 33.8) | 0.128 |
| Vegetal protein, % of energy | 5.60 (5.46; 5.73) | 5.62 (5.44, 5.79) | 5.57 (5.36, 5.79) | 0.738 |
| Vegetal protein-1000, g/d§ | 14.0 (13.6 14.3) | 14.1 (13.6, 14.5) | 13.9 (13.4, 14.5) | 0.738 |
| Vegetal protein, % of total protein | 45.7 (44.1, 47.3) | 46.5 (44.9, 48.2) | 44.9 (42.5, 47.3) | 0.274 |
| Vegetal protein-1000 / kg current weight, g/d | 0.28 (0.26, 0.29) | 0.27 (0.26, 0.28) | 0.29 (0.26, 0.31) | 0.255 |
| Vegetal protein-1000 / kg ideal theoretical weight, g/d | 0.28 (0.27, 0.30) | 0.28 (0.26, 0.29) | 0.29 (0.27, 0.31) | 0.274 |
| [n]  *Based on a 24-Hour Recall: Values are means and 95% CI, weighted for the survey design.  †*P* values for differences between men and women, as assessed by using Student´s t test.  ‡1 kcal/d = 4.18 kJ/d  §Energy-Adjusted by the density method [g / d per 1000 kcal] | | | | |

| **Table 5S** Total energy, animal, and vegetal protein intakes in the Colombian population (*18-64 y, non-pregnant women*). National Survey of Nutritional Situation in Colombia (ENSIN, 2015). | | | | |
| --- | --- | --- | --- | --- |
|  | All  [n = 10099] | Men  [n = 4801] | Women  [n = 5598] | *P*† |
|  | Mean (95% CI)* | Mean (95% CI)* | Mean (95% CI)* |  |
| Total energy, kcal/d‡ | 1999 (1964, 2035) | 2209 (2158, 2259) | 1814 (1768, 1859) | <0.0001 |
| Total protein, g/d | 65.4 (64.1, 66.8) | 71.3 (68.9, 73.7) | 60.2 (58.9, 61.6) | <0.0001 |
| Total protein-1000, g/d§ | 33.4 (32.7, 34.0) | 32.9 (31.8, 34.1) | 33.7 (33.2, 34.3) | 0.185 |
| Total protein, % of energy | 13.3 (13.1, 13.6) | 13.2 (12.7, 13.6) | 13.5 (13.3, 13.7) | 0.185 |
| Total protein / kg current weight, g/d | 0.99 (0.97, 1.01) | 1.02 (0.99, 1.06) | 0.96 (0.93, 0.99) | 0.011 |
| Total protein / kg ideal theoretical weight, g/d | 1.11 (1.01, 1.14) | 1.13 (1.09, 1.17) | 1.10 (1.07, 1.12) | 0.269 |
|  |  |  |  |  |
| Animal protein, g/d | 38.2 (37.3, 39.1) | 40.9 (39.3, 42.5) | 35.8 (34.9, 36.6) | <0.0001 |
| Animal protein, % of energy | 7.93 (7.72, 8.14 | 7.70 (7.32, 8.08) | 8.14 (7.93, 8.34) | 0.041 |
| Animal protein-1000, g/d§ | 19.8 (19.3, 20.4) | 19.3 (18.3, 20.2) | 20.3 (19.9, 20.8) | 0.041 |
| Animal protein, % of total protein | 58.1 (57.5, 58.7) | 57.1 (56.0, 58.2) | 59.0 (58.3, 59.7) | 0.007 |
| Animal protein-1000 / kg current weight, g/d | 0.30 (0.29, 0.31) | 0.28 (0.26, 0.29) | 0.32 (0.32, 0.33) | <0.0001 |
| Animal protein-1000 / kg ideal theoretical weight, g/d | 0.34 (0.33, 0.35) | 0.30 (0.29, 0.32) | 0.37 (0.36, 0.38) | <0.0001 |
|  |  |  |  |  |
| Vegetal protein, g/d | 27.1 (26.5, 27.7) | 29.7 (28.5, 37.8) | 24.8 (24.1, 25.5) | <0.0001 |
| Vegetal protein, % of energy | 5.47 (5.39, 5.56) | 5.41 (5.26, 5.56) | 5.53 (5.45, 5.61) | 0.144 |
| Vegetal protein-1000, g/d§ | 13.7 (13.5, 13.9) | 13.5 (13.2, 13.9) | 13.8 (13.6, 14.0) | 0.144 |
| Vegetal protein, % of total protein | 42.9 (42.2, 43.6) | 43.1 (41.9, 44.4) | 42.7 (42.0, 43.5) | 0.620 |
| Vegetal protein-1000 / kg current weight, g/d | 0.21 (0.20, 0.21) | 0.20 (0.19, 0.20) | 0.22 (0.22, 0.23) | <0.0001 |
| Vegetal protein-1000 / kg ideal theoretical weight, g/d | 0.24 (0.23, 0.24) | 0.21 (0.21, 0.22) | 0.25 (0.25, 0.26) | <0.0001 |
| [n]  *Based on a 24-Hour Recall: Values are means and 95% CI, weighted for the survey design.  †*P* values for differences between men and women, as assessed by using Student´s t test.  ‡1 kcal/d = 4.18 kJ/d  §Energy-Adjusted by the density method [g / d per 1000 kcal] | | | | |

| **Table 6S** Relative contribution (%)* of the main sources to the total protein in Colombian population (*1 to 64 y*). National Survey of Nutritional Situation in Colombia (ENSIN, 2015). | | | | | | |
| --- | --- | --- | --- | --- | --- | --- |
|  | | **%** | | | | |
| Food | All  [31135] | | 1 to 4 *y*  [6803] | 5 to 17 *y*  [14233] | 18 to 64 *y*  [10099] | Pregnant  Women  [1322] |
| Beef | 10.5 | | 7.5 | 10.4 | 11.6 | 13.0 |
| Cereals | 10.2 | | 9.2 | 11.2 | 10.2 | 6.0 |
| Bread, arepa, pasta | 10.2 | | 8.0 | 11.3 | 10.5 | 4.5 |
| Chicken meat (Poultry) | 9.7 | | 9.4 | 9.1 | 10.3 | 10.8 |
| Eggs | 6.3 | | 8.1 | 6.6 | 5.7 | 8.5 |
| Fish and shellfish | 5.6 | | 5.4 | 5.3 | 5.9 | 11.5 |
| Whole milk | 4.5 | | 10.1 | 4.6 | 3.5 | 5.6 |
| Non-canned processed meats | 4.4 | | 3.9 | 5.4 | 4.1 | 6.3 |
| Legumes and derived products | 4.0 | | 3.5 | 4.6 | 3.7 | 2.1 |
| Potatoes | 3.6 | | 4.0 | 3.3 | 3.8 | 2.2 |
| Cheese | 3.4 | | 3.1 | 3.8 | 3.4 | 4.9 |
| Pork Meat | 3.1 | | 1.7 | 2.9 | 3.8 | 4.3 |
| Organ meats and other cuts of beef, pork (Viscera) | 2.9 | | 2.6 | 2.8 | 1.0 | 7.4 |
| Derivatives of industrialized cereals | 2.3 | | 2.8 | 2.4 | 1.8 | 1.3 |
| Vegetables | 2.2 | | 2.1 | 1.8 | 2.4 | 0.5 |
| Fruit | 2.1 | | 2.5 | 1.6 | 2.1 | 1.2 |
| Cereal-based preparations, roots | 1.8 | | 0.8 | 2.2 | 1.8 | 0.7 |
| Banana preparations | 1.7 | | 2.0 | 1.7 | 1.7 | 1.7 |
| Homemade drinks with water and milk (Coffee) | 0.8 | | 0.5 | 0.8 | 1.0 | 0.0 |
| Homemade and industrialized desserts | 0.7 | | 0.6 | 0.7 | 0.5 | 0.2 |
| Fermented milks | 0.6 | | 1.5 | 0.6 | 0.3 | 0.8 |
| Roots, tubers | 0.6 | | 0.6 | 0.5 | 0.8 | 0.6 |
| Packaged foods (industrialized) | 0.5 | | 0.5 | 0.8 | 0.3 | 0.0 |
| Broths and soups | 0.5 | | 0.3 | 0.4 | 0.7 | 0.1 |
| Chocolate | 0.4 | | 0.3 | 0.4 | 0.3 | 0.0 |
| Vegetable mix | 0.4 | | 1.9 | 0.1 | 0.1 | 0.3 |
| Other meats | 0.4 | | 0.4 | 0.5 | 0.4 | 0.5 |
| Reduced fat milk | 0.3 | | 0.5 | 0.3 | 0.4 | 0.5 |
| Supplement and supplements | 0.3 | | 0.5 | 0.2 | 0.3 | 2.1 |
| [n]  * Calculated as [total protein per item / total protein] x 100 | | | | | | |

| **Table 7S** Sociodemographic characteristics of the Colombian population (*1* to *4* years, non-pregnant women) with estimates of the consumption of animal and vegetable protein, g/d for every 1000 kcal consumed, in the National Survey of Nutritional Situation in Colombia (ENSIN, 2015). | | | | | |
| --- | --- | --- | --- | --- | --- |
| Variable | Animal Protein* | | | Vegetal Protein* (Plants) | |
|  | n | Mean (g/d) † | SE | Mean (g/d) † | SE |
| Overall | 6923 | 20.8 | 02 | 12.9 | 0.4 |
| Sex  Males  Females | 3188  3735 | 21.2  20.5 | 0.2  0.3 | 13.2  12.6 | 0.1  0.6 |
| Height-for-age Z-score‡  <−2  −2 to<−1  −1 to 1  >1 to 2  >2 | 471  1252  2427  225  54 | 20.8  20.7  20.9  20.5  19.4 | 0.8  0.3  0.2  0.6  0.8 | 13.0  13.0  12.9  12.7  13.3 | 0.3  0.4  0.4  0.4  1.0 |
| BMI-for-age Z-score‡  <−2  −2 to<−1  −1 to 1  >1 to 2  >2 | 80  373  2922  788  265 | 21.1  20.8  21.0  20.3  20.4 | 0.9  0.5  0.3  0.4  0.5 | 12.2  13.3  13.0  12.6  12.9 | 0.6  0.4  0.3  0.4  0.5 |
| Education of head  <5 (Primary or less)  5 to <11  11 to <16  ≥16 (University) | 1840  2392  2284  364 | 20.3  21.1  20.8  20.6 | 0.4  0.3  0.3  0.5 | 13.1  12.9  12.7  12.8 | 0.3  0.3  0.6  0.5 |
| Wealth index, quintiles§  Q1  Q2  Q3  Q4 | 3766  1683  923  551 | 19.9  20.5  22.7  23.0 | 0.2  0.4  0.4  0.9 | 12.5  13.0  13.0  14.4 | 0.6  0.2  0.2  1.0 |
| Food insecurity in the home  No  Mild  Moderate  Severe | 2334  2530  1178  880 | 20.9  21.1  20.7  19.7 | 0.3  0.3  0.3  0.6 | 12.8  12.9  12.8  13.1 | 0.4  0.4  0.5  0.4 |
| Urbanicity  Big cities\|\|  100001 a 1000000 population  0 a 100000 population  Disperse population | 1047  1572  2370  1934 | 21.3  20.9  21.2  19.9 | 0.7  0.5  0.4  0.3 | 12.4  13.1  12.6  13.5 | 1.0  0.2  0.2  0.1 |
| Country region  Central  Atlantic (North)  Oriental  Pacific (West)  Bogotá  Amazonia-Orinoquia | 1399  1536  1269  950  454  1315 | 20.2  19.9  21.7  20.7  23.1  22.2 | 0.4  0.3  0.3  0.4  0.6  0.8 | 12.6  12.4  13.5  13.3  13.4  12.7 | 0.3  0.9  0.1  0.2  0.3  0.2 |
| *Based on 24-Hour Recall.  †Energy-Adjusted by the density method. Grams/day for every 1000 kcal consumed: 1 kcal/d = 4.18 kJ/d.  ‡According to the WHO.^25^  §The wealth index is a composite measure of a household’s cumulative living standard. The wealth index is calculated using easy-to-collect data on a household’s ownership of selected assets such as televisions and bicycles, materials used for housing construction, type of water supply and sanitation facilities.^21^  \|\|Bogotá, Barranquilla, Medellín, Cali y Bucaramanga. | | | | | |

| **Table 8S** Differences in animal protein intake, g/d for every 1000 kcal consumed, in Colombian population (*1 to 4 years, non-pregnant women*) according to sociodemographic characteristics. National Survey of Nutritional Situation in Colombia (ENSIN, 2015). | | | | |
| --- | --- | --- | --- | --- |
| Variable | Crude difference* (95% CI) | *P*† | Adjusted difference‡ (95% CI) | *P*§ |
| Sex  Males  Females | -  -0.7 (-1.5, 0.0) | 0.051 | -  -0.3 (-1.1, 0.4) | 0.347 |
| Height-for-age Z-score\|\|  <−2  −2 to<−1  −1 to 1  >1 to 2  >2 | -0.1 (-1.6, 1.4)  -0.2 -0.8, 0.3)  -  -0.4 (-1.7, 0.9)  -1.5 (-3.1, 0.1) | 0.908 | -0.3 (-1.6, 0.9)  -0.2 (-0.7, 0.4)  -  -0.4 (-1.8, 0.9)  -1.0 (-2.6, 0.6) | 0.872 |
| BMI-for-age Z-score\|\|  <−2  −2 to<−1  −1 to 1  >1 to 2  >2 | 0.1 (-1.6, 1.8)  -0.2 (-1.3, 0.8)  -  -0.7 (-1.5, 0.2)  -0.6 (-1.7, 0.4) | 0.195 | 0.4 (-1.2, 2.0)  -0.3 (-1.2, 0.7)  -  -0.7 (-1.5, 0.1)  -0.6 (-1.7, 0.4) | 0.162 |
| Education of head  <5 (Primary or less)  5 to <11  11 to <16  ≥16 (University) | -0.8 (-1.6, -0.0)  -  -0.3 (-0.9, 0.3)  -0.5 (-1.6, 0.6) | 0.406 | -0.5 (-1.3, 0.2)  -  -0.3 (-0.9, 0.3)  -0.5 (-1.6, 0.5) | 0.866 |
| Wealth index, quintiles¶  Q1  Q2  Q3  Q4 | -3.2 (-5.0, -1.3)  -2.5 (-4.6, -0.5)  -0.4 (-2.4, 1.6)  - | <0.0001 | -3.0 (-4.6, -1.4)  -2.6 (-4.4, -0.8)  -0.7 (-2.4, 1.1)  - | <0.0001 |
| Food insecurity  No  Mild  Moderate  Severe | -  0.2 (-0.5, 0.9)  -0.2 (-0.9, 0.5)  -1.2 (-2.5, -0.0) | 0.066 | -  0.3 (-0.4, 0.9)  0.2 (-0.5, 0.8)  -0.9 (-2.0, 0.3) | 0.250 |
| Urbanicity  Big cities**  100001 a 1000000 population  0 a 100000 population  Disperse population | -  -0.4 (-2.1, 1.3)  -0.1 (-1.6, 1.4)  -1.4 (-2.8, 0.1) | 0.113 | -  -0.3 (-1.5, 0.9)  0.3 (-0.6, 1.1)  -0.1 (-1.0, 0.8) | 0.995 |
| Country region  Central  Atlantic (North)  Oriental  Pacific (West)  Bogotá  Amazonia-Orinoquia | -  -0.2 (-1.2, 0.7)  1.5 (0.5, 2.4)  0.6 (-0.6, 1.7)  2.9 (1.5, 4.3)  2.0 (0.4, 3.7) | <0.0001 | -  0.2 (-0.9, 1.3)  1.5 (0.5, 2.4)  1.0 (-0.2, 2.1)  2.2 (0.7, 3.6)  2.5 (0.9, 4.1) | <0.0001 |
| *Based on 24-Hour Recall. Energy-Adjusted by the density method. Grams/day for every 1000 kcal consumed: 1 kcal/d = 4.18 kJ/d.  Test for linear trend for ordinal predictors. For sex, urbanicity and country region, *P* is from ANOVA. All tests incorporated the complex sampling survey design.  ‡From linear regression models with protein intake as continuous result and indicator variables in the table as predictors except for Height-for-age and BMI-for-age. The estimates for education come from a model that excludes the wealth index and food security, which could be on the causal path. The wealth index estimates excluded food security.  §Adjusted test for linear trend or ANOVA for ordinal or categorical correlates, respectively.  \|\|According to the WHO.^25^  ¶The wealth index is a composite measure of a household’s cumulative living standard. The wealth index is calculated using easy-to-collect data on a household’s ownership of selected assets such as televisions and bicycles, materials used for housing construction, type of water supply and sanitation facilities.^21^  **Bogotá, Barranquilla, Medellín, Cali y Bucaramanga. | | | | |

| **Table 9S** Differences in vegetal (Plants) protein intake, g/d for every 1000 kcal consumed, in Colombian population (*1 to 4 years, non-pregnant women*) according to sociodemographic characteristics. National Survey of Nutritional Situation in Colombia (ENSIN, 2015). | | | | |
| --- | --- | --- | --- | --- |
| Variable | Crude difference* (95% CI) | *P*† | Adjusted difference‡ (95% CI) | *P*§ |
| Sex  Males  Females | -  -0.6 (-1.9, 0.7) | 0.371 | -  -0.2 (-0.8, 0.5) | 0.615 |
| Height-for-age Z-score\|\|  <−2  −2 to<−1  −1 to 1  >1 to 2  >2 | 0.1 (-0.5, 0.8)  0.2 (-0.2, 0.5)  -  -0.2 (-0.6, 0.3)  -0.2 (-0.6, 0.3) | 0.514 | 0.0 (-0.6, 0.6)  0.1 (-0.2, 0.5)  -  -0.1 (-0.1, 0.4)  0.8 (-0.4, 2.0) | 1.0 |
| BMI-for-age Z-score\|\|  <−2  −2 to<−1  −1 to 1  >1 to 2  >2 | -0.8 (-1.6, 0.0)  0.3 (-0.1, 0.7)  -  -0.4 (-0.7, -0.1)  -0.1 (-0.6, 0.4) | 0.196 | -0.6 (-1.4, 0.1)  0.2 (-0.2, 0.6)  -  -0.3 (-0.6, -0.0)  -0.0 (-0.6, 0.5) | 0.389 |
| Education of head  <5 (Primary or less)  5 to <11  11 to <16  ≥16 (University) | 0.2 (-0.2, 0.6)  -  -0.1 (-0.8, 0.5)  -0.1 (-0.6, 0.4) | 0.353 | 0.1 (-0.2, 052)  -  0.0 (-0.3, 0.4)  0.0 (-0.5, 0.5) | 0.628 |
| Wealth index, quintiles¶  Q1  Q2  Q3  Q4 | -1.8 (-4.1,0.4)  -1.4 (-3.3, 0.6)  -1.4 (-3.3, 0.6)  - | 0.161 | -2.5 (-5.0, -0.1)  -1.8 (-4.0, 0.4)  -1.7 (-3.8, 0.5)  - | 0.046 |
| Food insecurity  No  Mild  Moderate  Severe | -  0.6 (-0.3, 0.4)  -0.0 (-0.4, 0.3)  0.3 (-0.2, 0.7) | 0.390 | -  0.1 (-0.3, 0.5)  0.1 (0.3, 0.5)  0.5 (-0.1, 0.1) | 0.139 |
| Urban City  Big cities**  100001 a 1000000 population  0 a 100000 population  Disperse population | -  0.7 (-1.3, 2.7)  0.2 (-1.7, 2.2)  1.1 (-0.9, 3.0) | 0.362 | -  0.7 (-0.7, 2.0)  0.5 (-0.7, 1.6)  1.9 (0.6, 3.2) | 0.012 |
| Country region  Central  Atlantic (North)  Oriental  Pacific (West)  Bogotá  Amazonia-Orinoquia | -  -0.2 (-2.2, 1.7)  1.0 (0.3, 1.5)  0.6 (-0.1, 1.3)  0.7 (0.0, 1.5)  0.1 (-0.5, 0.7) | 0.156 | -  0.5 (-0.6, 1.7)  0.8 (0.2, 1.3)  0.7 (0.0, 1.4)  1.3 (0.3, 2.3)  0.1 (-0.5, 0.8) | 0.068 |
| *Based on 24-Hour Recall. Energy-Adjusted by the density method. Grams/day for every 1000 kcal consumed: 1 kcal/d = 4.18 kJ/d.  Test for linear trend for ordinal predictors. For sex, urbanicity and country region, *P* is from ANOVA. All tests incorporated the complex sampling survey design.  ‡From linear regression models with protein intake as continuous result and indicator variables in the table as predictors except for Height-for-age and BMI-for-age. The estimates for education come from a model that excludes the wealth index and food security, which could be on the causal path. The wealth index estimates excluded food security.  §Adjusted test for linear trend or ANOVA for ordinal or categorical correlates, respectively.  \|\|According to the WHO.^25^  ¶The wealth index is a composite measure of a household’s cumulative living standard. The wealth index is calculated using easy-to-collect data on a household’s ownership of selected assets such as televisions and bicycles, materials used for housing construction, type of water supply and sanitation facilities.^21^  **Bogotá, Barranquilla, Medellín, Cali y Bucaramanga. | | | | |

| **Table 10S** Sociodemographic characteristics of the Colombian population (*5 to 12 years, non-pregnant women*) with estimates of the consumption of animal and vegetable protein, g/d for every 1000 kcal consumed, in the National Survey of Nutritional Situation in Colombia (ENSIN, 2015). | | | | | |
| --- | --- | --- | --- | --- | --- |
| Variable | Animal Protein* | | | Vegetal Protein* (Plants) | |
|  | n | Mean (g/d) † | SE | Mean (g/d) † | SE |
| Overall | 6692 | 18.2 | 0.5 | 14.7 | 0.7 |
| Sex  Males  Females | 3782  2910 | 17.8  18.9 | 0.7  0.3 | 15.2  14.0 | 0.9  0.3 |
| Height-for-age Z-score‡  <−2  −2 to<−1  −1 to 1  >1 to 2  >2 | 471  1236  2486  225  50 | 18.4  18.4  18.4  18.8  18.2 | 0.5  0.7  0.6  0.7  1.2 | 14.9  14.9  14.5  14.3  15.1 | 0.7  0.5  0.6  0.7  0.8 |
| BMI-for-age Z-score‡  <−2  −2 to<−1  −1 to 1  >1 to 2  >2 | 73  373  2947  787  273 | 17.8  18.1  18.4  18.1  19.1 | 0.9  0.6  0.5  0.5  1.0 | 15.1  14.9  14.5  14.7  14.9 | 0.8  0.6  0.6  0.6  0.8 |
| Education of head  <5 (Primary or less)  5 to <11  11 to <16  ≥16 (University) | 1782  2300  2155  375 | 17.8  18.2  18.5  18.0 | 0.5  0.6  0.7  0.6 | 14.7  15.0  14.7  14.1 | 0.6  0.7  0.7  0.7 |
| Wealth index, quintiles§  Q1  Q2  Q3  Q4 | 3711  1439  1063  479 | 16.9  18.3  20.4  21.0 | 0.3  0.5  0.6  1.1 | 15.6  14.4  13.9  12.6 | 0.9  0.3  0.2  0.4 |
| Food insecurity in the home  No  Mild  Moderate  Severe | 2374  2309  1118  816 | 18.2  18.4  17.8  18.0 | 0.7  0.6  0.5  0.5 | 14.9  14.5  14.5  15.3 | 0.8  0.6  0.5  0.8 |
| Urban City  Big cities\|\|  100001 a 1000000 population  0 a 100000 population  Disperse population | 1326  1221  2724  1421 | 17.9  19.1  18.5  17.7 | 1.0  0.7  0.4  0.4 | 15.8  13.9  13.6  14.1 | 0.9  0.4  0.1  0.5 |
| Country region  Central  Atlantic (North)  Oriental  Pacific (West)  Bogotá  Amazonia-Orinoquia | 1777  831  1046  608  627  1803 | 17.0  17.7  19.0  17.6  20.5  21.1 | 0.6  0.5  0.4  0.6  0.8  0.9 | 15.9  13.9  13.9  14.4  14.1  13.1 | 1.0  0.6  0.2  0.4  0.2  0.2 |
| *Based on 24-Hour Recall.  †Energy-Adjusted by the density method. Grams/day for every 1000 kcal consumed: 1 kcal/d = 4.18 kJ/d.  ‡According to the WHO.^25^  §The wealth index is a composite measure of a household’s cumulative living standard. The wealth index is calculated using easy-to-collect data on a household’s ownership of selected assets such as televisions and bicycles, materials used for housing construction, type of water supply and sanitation facilities.^21^  \|\|Bogotá, Barranquilla, Medellín, Cali y Bucaramanga. | | | | | |

| **Table 11S** Differences in animal protein intake, g/d for every 1000 kcal consumed, in Colombian population (5 *to 12 years, non-pregnant women*) according to sociodemographic characteristics. National Survey of Nutritional Situation in Colombia (ENSIN, 2015). | | | | |
| --- | --- | --- | --- | --- |
| Variable | Crude difference* (95% CI) | *P*† | Adjusted difference‡ (95% CI) | *P*§ |
| Sex  Males  Females | -  1.09 (-0.4, 2.6) | 0.159 | -  0.1 (-0.9, 1.1) | 0.841 |
| Height-for-age Z-score\|\|  <−2  −2 to<−1  −1 to 1  >1 to 2  >2 | 0.0 (-1.0, 1.0)  0.0 -0.9, 1.0)  -  0.5 (-0.6, 0.5)  -0.1 (-2.2, 1.9) | 0.847 | -0.1 (-1., 0.8)  0.1 (-0.8, 0.9)  -  0.3 (-0.8, 1.4)  -0.1 (-2.0, 1.8) | 0.853 |
| BMI-for-age Z-score\|\|  <−2  −2 to<−1  −1 to 1  >1 to 2  >2 | -0.6 (-2.4, 1.1)  -0.3 (-1.2, 0.6)  -  -0.3 (-1.0, 0.4)  0.7 (-0.7, 2.0) | 0.384 | 0.1 (-1.7, 1.9)  -0.4 (-0.8, 0.8)  -  -0.2 (-0.9, 0.4)  1.0 (-0.1, 2.1) | 0.409 |
| Education of head  <5 (Primary or less)  5 to <11  11 to <16  ≥16 (University) | -0.4 (-1.3, 0.5)  -  0.3 (-0.3, 1.0)  -0.1 (-1.3, 1.0) | 0.124 | -0.2 (-1.0, 0.5)  -  0.3 (-0.3, 1.0)  -0.3 (-1.3, 0.8) | 0.231 |
| Wealth index, quintiles¶  Q1  Q2  Q3  Q4 | -4.1 (-6.4, -1.8)  -2.7 (-5.1, -0.3)  -0.6 (-3.1, 1.9)  - | <0.0001 | -3.8 (-6.5, -1.0)  -2.9 (-5.4, -0.4)  -0.9 (-3.3, 1.5)  - | 0.001 |
| Food insecurity  No  Mild  Moderate  Severe | -  0.3 (-0.4, 0.9)  -0.4 (-1.5, 0.7)  -0.1 (-1.2, 0.9) | 0.636 | -  0.2 (-0.4, 0.8)  -0.5 (-1.4, 0.4)  0.1 (-0.8, 0.9) | 0.627 |
| Urban City  Big cities**  100001 a 1000000 population  0 a 100000 population  Disperse population | -  1.2 (-1.2, 3.6)  0.6 (-1.5, 2.7)  -0.1 (-2.2, 1.9) | 0.968 | -  0.4 (-1.0, 1.7)  0.5 (-5.0, 1.6)  0.8 (-0.5, 2.0) | 0.205 |
| Country region  Central  Atlantic (North)  Oriental  Pacific (West)  Bogotá  Amazonia-Orinoquia | -  0.7 (-0.9, 2.3)  1.9 (0.5, 3.4)  0.6 (-1.1, 2.3)  3.5 (1.5, 5,5)  4.1 (1.9, 6.2) | <0.0001 | -  -0.1 (-1.5, 1.4)  0.4 (-1.2, 2.0)  -0.1 (-1.7, 1.4)  1.9 (-0.2, 4.0)  3.1 (1.1, 5.1) | 0.029 |
| *Based on 24-Hour Recall. Energy-Adjusted by the density method. Grams/day for every 1000 kcal consumed: 1 kcal/d = 4.18 kJ/d.  Test for linear trend for ordinal predictors. For sex, urbanicity and country region, *P* is from ANOVA. All tests incorporated the complex sampling survey design.  ‡From linear regression models with protein intake as continuous result and indicator variables in the table as predictors except for Height-for-age and BMI-for-age. The estimates for education come from a model that excludes the wealth index and food security, which could be on the causal path. The wealth index estimates excluded food security.  §Adjusted test for linear trend or ANOVA for ordinal or categorical correlates, respectively.  \|\|According to the WHO.^25^  ¶The wealth index is a composite measure of a household’s cumulative living standard. The wealth index is calculated using easy-to-collect data on a household’s ownership of selected assets such as televisions and bicycles, materials used for housing construction, type of water supply and sanitation facilities.^21^  **Bogotá, Barranquilla, Medellín, Cali y Bucaramanga. | | | | |

| **Table 12S** Differences in vegetal (Plants) protein intake, g/d for every 1000 kcal consumed, in Colombian population (*5 to 12 years, non-pregnant women*) according to sociodemographic characteristics. National Survey of Nutritional Situation in Colombia (ENSIN, 2015). | | | | |
| --- | --- | --- | --- | --- |
| Variable | Crude difference* (95% CI) | *P*† | Adjusted difference‡ (95% CI) | *P*§ |
| Sex  Males  Females | -  -1.2 (-3.1, 0.6) | 0.172 | -  -0.3 (-0.8, 0.3) | 0.331 |
| Height-for-age Z-score\|\|  <−2  −2 to<−1  −1 to 1  >1 to 2  >2 | 0.3 (-1.1, 1.8)  0.3 (-0.2, 0.9)  -  -0.2 (-1.0, 0.6)  0.5 (-0.8, 1.9) | 0.400 | 0.4 (-0.5, 1.3)  0.3 (-0.1, 0.7)  -  -0.1 (-0.8, 0.5)  0.6 (-0.7, 1.9) | 0.183 |
| BMI-for-age Z-score\|\|  <−2  −2 to<−1  −1 to 1  >1 to 2  >2 | 0.5 (-0.4, 1.4)  0.4 (-0.0, 0.8)  -  0.2 (-0.3, 0.6)  0.4 (-0.3, 1.1) | 0.978 | - 1. (-0.7, 0.9)   0.1 (-0.3, 0.5)  0.0 (-0.4, 0.5)  0.1 (-0.4, 0.5) | 0.965 |
| Education of head  <5 (Primary or less)  5 to <11  11 to <16  ≥16 (University) | -0.3 (-1.0, 0.4)  -  -0.3 (-0.6, 0.1)  -0.9 (-1.9, 0.1) | 0.493 | 0.1 (-0.6, 0.7)  -0.3 (-0.6, 0.0)  -  -0.8 (-1.8, 0.2) | 0.076 |
| Wealth index, quintiles¶  Q1  Q2  Q3  Q4 | 3.0 (1.0, 5.0)  1.8 (0.8, 2.9)  1.4 (0.5, 2.3)  - | 0.016 | 3.3 (2.1, 4.5)  2.2 (1.1, 3.2)  1.5 (0.6, 2.4)  - | <0.0001 |
| Food insecurity  No  Mild  Moderate  Severe | -  -0.4 (-0.9, -0.0)  -0.5 (-1.3, 0.3)  0.4 (-1.3, 2.0) | 0.952 | -  -0.3 (-0.6, -0.0)  -0.2 (-0.7, 0.2)  0.6 (-0.7, 1.9) | 0.516 |
| Urban City  Big cities**  100001 a 1000000 population  0 a 100000 population  Disperse population | -  -1.9 (-3.8, 0.1)  -2.2 (-4.0, -0.3)  -1.7 (-3.7, 0.4) | 0.116 | -  -1.3 (-2.1, -0.4)  -2.1 (-2.8, -1.4)  -2.3 (-3.3, -1.4) | <0.0001 |
| Country region  Central  Atlantic (North)  Oriental  Pacific (West)  Bogotá  Amazonia-Orinoquia | -  -2.1 (-4.3, 0.2)  -2.0 (-4.0, -0.1)  -1.6 (-3.6, 0.5)  -1.8 (-3.7, 0.2)  -2.9 (-4.8, -0.9) | 0.126 | -  -1.2 (-2.3, -0.1)  -0.3 (-1.0, 0.5)  -0.5 (-1.6, 0.7)  -0.9 (-1.7, -0.0)  -1.5 (-2.2, -0.7) | 0.049 |
| *Based on 24-Hour Recall. Energy-Adjusted by the density method. Grams/day for every 1000 kcal consumed: 1 kcal/d = 4.18 kJ/d.  Test for linear trend for ordinal predictors. For sex, urbanicity and country region, *P* is from ANOVA. All tests incorporated the complex sampling survey design.  ‡From linear regression models with protein intake as continuous result and indicator variables in the table as predictors except for Height-for-age and BMI-for-age. The estimates for education come from a model that excludes the wealth index and food security, which could be on the causal path. The wealth index estimates excluded food security.  §Adjusted test for linear trend or ANOVA for ordinal or categorical correlates, respectively.  \|\|According to the WHO.^25^  ¶The wealth index is a composite measure of a household’s cumulative living standard. The wealth index is calculated using easy-to-collect data on a household’s ownership of selected assets such as televisions and bicycles, materials used for housing construction, type of water supply and sanitation facilities.^21^  **Bogotá, Barranquilla, Medellín, Cali y Bucaramanga. | | | | |

| **Table 13S** Sociodemographic characteristics of the Colombian population (*13 to 17 years, non-pregnant women*) with estimates of the consumption of animal and vegetable protein, g/d for every 1000 kcal consumed, in the National Survey of Nutritional Situation in Colombia (ENSIN, 2015). | | | | | |
| --- | --- | --- | --- | --- | --- |
| Variable | Animal Protein* | | | Vegetal Protein* (Plants) | |
|  | n | Mean (g/d) † | SE | Mean (g/d) † | SE |
| Overall | 7541 | 17.8 | 0.5 | 14.0 | 0-2 |
| Sex  Males  Females | 3545  3996 | 17.0  18.4 | 0.4  0.7 | 14.1  13.9 | 0.2  0.3 |
| Height-for-age Z-score‡  <−2  −2 to<−1  −1 to 1  >1 to 2  >2 | 499  1371  2678  260  69 | 17.3  17.4  18.1  18.8  17.0 | 0.6  0.5  0.6  0.8  1.5 | 14.0  13.9  13.9  14.1  14.1 | 0.3  0.2  0.2  0.3  0.5 |
| BMI-for-age Z-score‡  <−2  −2 to<−1  −1 to 1  >1 to 2  >2 | 80  455  3167  884  293 | 16.7  17.6  17.8  18.1  18.0 | 0.9  0.6  0.5  0.8  0.7 | 14.2  13.8  14.0  14.0  14.1 | 0.6  0.3  0.2  0.3  0.3 |
| Education of head  <5 (Primary or less)  5 to <11  11 to <16  ≥16 (University) | 2201  2556  2366  378 | 17.5  17.4  18.0  20.1 | 0.5  0.5  0.6  1.6 | 13.8  14.1  14.1  13.6 | 0.2  0.2  0.2  0.5 |
| Wealth index, quintiles§  Q1  Q2  Q3  Q4 | 3614  2069  1214  644 | 15.5  18.5  18.4  20.8 | 0.4  1.0  0.7  0.7 | 13.8  14.5  13.9  13.4 | 0.1  0.4  0.2  0.3 |
| Food insecurity in the home  No  Mild  Moderate  Severe | 2520  2750  1362  907 | 18.6  17.4  17.0  17.4 | 0.6  0.5  0.5  0.7 | 14.1  13.9  14.1  13.7 | 0.2  0.2  0.2  0.3 |
| Urban City  Big cities\|\|  100001 a 1000000 population  0 a 100000 population  Disperse population | 1022  2027  2631  1861 | 19.8  17.4  17.4  16.2 | 0.8  0.8  0.4  0.3 | 14.7  13.8  13.6  13.8 | 0.2  0.4  0.3  0.2 |
| Country region  Central  Atlantic (North)  Oriental  Pacific (West)  Bogotá  Amazonia-Orinoquia | 1770  1452  1257  1388  421  1253 | 16.7  16.6  19.5  17.9  19.4  17.3 | 0.4  0.7  0.6  1.5  0.9  0.6 | 13.6  13.2  14.0  14.8  14.7  13.4 | 0.2  0.3  0.2  0.4  0.2  0.2 |
| *Based on 24-Hour Recall.  †Energy-Adjusted by the density method. Grams/day for every 1000 kcal consumed: 1 kcal/d = 4.18 kJ/d.  ‡According to the WHO.^25^  §The wealth index is a composite measure of a household’s cumulative living standard. The wealth index is calculated using easy-to-collect data on a household’s ownership of selected assets such as televisions and bicycles, materials used for housing construction, type of water supply and sanitation facilities.^21^  \|\|Bogotá, Barranquilla, Medellín, Cali y Bucaramanga. | | | | | |

| **Table 14S** Differences in animal protein intake, g/d for every 1000 kcal consumed, in Colombian population (*13 to 17 years, non-pregnant women*) according to sociodemographic characteristics. National Survey of Nutritional Situation in Colombia (ENSIN, 2015). | | | | |
| --- | --- | --- | --- | --- |
| Variable | Crude difference* (95% CI) | *P*† | Adjusted difference‡ (95% CI) | *P*§ |
| Sex  Males  Females | -  1.3 (-0.3, 3.0) | 0.103 | -  1.5 (0.2, 2.8) | 0.025 |
| Height-for-age Z-score\|\|  <−2  −2 to<−1  −1 to 1  >1 to 2  >2 | -0.8 (-2.0, 0.5)  -0.7 -1.7, 0.3)  -  0.7 (-0.7, 2.1)  -1.1 (-3.8, 1.6) | 0.117 | -0.2 (-1.2, 0.9)  -0.3 (-1.1, 0.5)  -  0.6 (-0.7, 1.8)  1.0 (-3.7, 1.7) | 0.581 |
| BMI-for-age Z-score\|\|  <−2  −2 to<−1  −1 to 1  >1 to 2  >2 | -1.1 (-2.9, 0.6)  -0.2 (-1.3, 0.8)  -  0.2 (-1.0, 1.4)  0.2 (-1.0, 1.3) | 0.320 | -0.6 (-2.5, 1.2)  -0.1 (-1.1, 0.8)  -  -0.3 (-1.1, 0.6)  0.1 (-1.1, 1.2) | 0.964 |
| Education of head  <5 (Primary or less)  5 to <11  11 to <16  ≥16 (University) | 0.2 (-0.7, 1.1)  -  0.6 (-0.1, 1.3)  2.7 (-0.5, 5.9) | 0.087 | 0.7 (-0.2, 1.7)  -  0.4 (-0.2, 1.1)  2.6 (-0.4, 5.6) | 0.503 |
| Wealth index, quintiles¶  Q1  Q2  Q3  Q4 | -5.4 (-7.1, -3.6)  -2.4 (-4.8, 0.1)  -2.4 (-4.4, -0.4)  - | <0.0001 | -5,5 (-7.9, -3.0)  -2.5 (-4.7, -0.3)  -2.3 (-4.3, -0.3)  - | <0.0001 |
| Food insecurity  No  Mild  Moderate  Severe | -  -1.1 (-2.1, -0.1)  -1-6 (-2.6, -0.5)  -1.2 (-2.5, 0.1) | 0.016 | -  -1.0 (-2.0, -0.1)  -1.0 (-1.9, -0.0)  -0.4 (-1.5, 0.8) | 0.207 |
| Urban City  Big cities**  100001 a 1000000 population  0 a 100000 population  Disperse population | -  -2.4 (-4.6, -0.2)  -2.4 (-4.2, -0.7)  -3.6 (-5.4, -1.9) | 0.001 | -  -1.6 (-4.0, 0.8)  -0.6 (-2.9, 1.7)  -0.6 (-3.3, 2.2) | 0.937 |
| Country region  Central  Atlantic (North)  Oriental  Pacific (West)  Bogotá  Amazonia-Orinoquia | -  -0.1 (-1.8, 1.6)  2.8 (1.3, 4.3)  1.2 (-1.9, 4.3)  2.7 (0.7, 4.7)  0.6 (-0.9, 2.1) | 0.034 | -  0.1 (-1.4, 1.6)  2.3 (1.1, 3.5)  0.9 (-1.3, 3.0)  0.7 (-1.8, 3.1)  1.4 (0.0, 2.7) | 0.127 |
| *Based on 24-Hour Recall. Energy-Adjusted by the density method. Grams/day for every 1000 kcal consumed: 1 kcal/d = 4.18 kJ/d.  Test for linear trend for ordinal predictors. For sex, urbanicity and country region, *P* is from ANOVA. All tests incorporated the complex sampling survey design.  ‡From linear regression models with protein intake as continuous result and indicator variables in the table as predictors except for Height-for-age and BMI-for-age. The estimates for education come from a model that excludes the wealth index and food security, which could be on the causal path. The wealth index estimates excluded food security.  §Adjusted test for linear trend or ANOVA for ordinal or categorical correlates, respectively.  \|\|According to the WHO.^25^  ¶The wealth index is a composite measure of a household’s cumulative living standard. The wealth index is calculated using easy-to-collect data on a household’s ownership of selected assets such as televisions and bicycles, materials used for housing construction, type of water supply and sanitation facilities.^21^  **Bogotá, Barranquilla, Medellín, Cali y Bucaramanga. | | | | |

| **Table 15S** Differences in vegetal (Plants) protein intake, g/d for every 1000 kcal consumed, in Colombian population (*13 to 17 years, non-pregnant women*) according to sociodemographic characteristics. National Survey of Nutritional Situation in Colombia (ENSIN, 2015). | | | | |
| --- | --- | --- | --- | --- |
| Variable | Crude difference* (95% CI) | *P*† | Adjusted difference‡ (95% CI) | *P*§ |
| Sex  Males  Females | -  -0.1 (-0.8, 0.6) | 0.103 | -  -0.3 (-0.8, 0.3) | 0.318 |
| Height-for-age Z-score\|\|  <−2  −2 to<−1  −1 to 1  >1 to 2  >2 | 0.0 (-0.5, 0.6)  -0.0 (-0.4, 0.3)  -  0.2 (-0.4, 0.7)  0.2 (-0.8, 1.1) | 0.805 | 0.0 (-0.5, 0.6)  -0.0 (-0.3, 0.3)  -  0.2 (-0.3, 0.7)  0.1 (-0.8, 1.0) | 0.768 |
| BMI-for-age Z-score\|\|  <−2  −2 to<−1  −1 to 1  >1 to 2  >2 | 0.2 (-0.9, 1.3)  -0.2 (-0.7, 0.3)  -  -0.0 (-0.3, 0.3)  0.1 (-0.3, 0.6) | 0.633 | 0.1 (-0.9, 1.1)  -0.2 (-0.6, 0.3)  0.0 (-0.3, 0.3)  0.2 (-0.2, 0.7) | 0.405 |
| Education of head  <5 (Primary or less)  5 to <11  11 to <16  ≥16 (University) | -0.3 (-0.6, 0.1)  -  -0.0 (-0.4, 0.3)  -0.5 (-1.5, 0.4) | 0.750 | -0.1 (-0.4, 0.2)  -0.1 (-0.4, 0.2)  -  -0.5 (-1.4, 0.4) | 0.483 |
| Wealth index, quintiles¶  Q1  Q2  Q3  Q4 | 0.4 (-0.2, 1.1)  1.1 (0.1, 2.1)  0.5 (-0.2, 1.2)  - | 0.226 | 1.1 (0.4, 1.8)  1.2 (0.4, 2.1)  0.6 (-0.0, 1.3)  - | 0.001 |
| Food insecurity  No  Mild  Moderate  Severe | -  -0.1 (-0.5, 0.2)  0.0 (-0.4, 0.4)  -0.3 (-0.9, 0.2) | 0.373 | -  -0.1 (-0.4, 0.2)  0.1 (-0.3, 0.5)  -0.3 (-0.9, 0.2) | 0.447 |
| Urban City  Big cities**  100001 a 1000000 population  0 a 100000 population  Disperse population | -  -0.9 (-1.7, -0.1)  -1.1 (-1.7, -0.4)  -0.9 (-1.4, -0.4) | 0.017 | -  -0.6 (-1.5, 0.2)  -1.1 (-1.6, -0.5)  -1.1 (-1.8, -0.5) | 0.002 |
| Country region  Central  Atlantic (North)  Oriental  Pacific (West)  Bogotá  Amazonia-Orinoquia | -  -0.4 (-1.1, 0.3)  0.4 (-0.2, 1.0)  1.2 (0.4, 2.0)  1.1 (0.5, 1.6)  -0.2 (-0.7, 0.2) | <0.0001 | -  -0.5 (-1.2, 0.1)  0.6 (0.0, 1.1)  1.0 (0.3, 1.7)  0.9 (0.2, 1.6)  -0.3 (-0.8, 0.2) | 0.001 |
| *Based on 24-Hour Recall. Energy-Adjusted by the density method. Grams/day for every 1000 kcal consumed: 1 kcal/d = 4.18 kJ/d.  Test for linear trend for ordinal predictors. For sex, urbanicity and country region, *P* is from ANOVA. All tests incorporated the complex sampling survey design.  ‡From linear regression models with protein intake as continuous result and indicator variables in the table as predictors except for Height-for-age and BMI-for-age. The estimates for education come from a model that excludes the wealth index and food security, which could be on the causal path. The wealth index estimates excluded food security.  §Adjusted test for linear trend or ANOVA for ordinal or categorical correlates, respectively.  \|\|According to the WHO.^25^  ¶The wealth index is a composite measure of a household’s cumulative living standard. The wealth index is calculated using easy-to-collect data on a household’s ownership of selected assets such as televisions and bicycles, materials used for housing construction, type of water supply and sanitation facilities.^21^  **Bogotá, Barranquilla, Medellín, Cali y Bucaramanga. | | | | |

| **Table 16S** Sociodemographic characteristics of the Colombian population (*18 to 64 years, non-pregnant women*) with estimates of the consumption of animal and vegetable protein, g/d for every 1000 kcal consumed, in the National Survey of Nutritional Situation in Colombia (ENSIN, 2015). | | | | | |
| --- | --- | --- | --- | --- | --- |
| Variable | Animal Protein* | | | Vegetal Protein* (Plants) | |
|  | n | Mean (g/d) † | SE | Mean (g/d) † | SE |
| Overall | 10099 | 19.8 | 0.3 | 13.7 | 0.1 |
| Sex  Males  Females | 4801  5298 | 19.3  20.3 | 0.5  0.2 | 13.5  13.8 | 0.2  0.1 |
| Education of head  <5 (Primary or less)  5 to <11  11 to <16  ≥16 (University) | 2880  3386  3203  551 | 18.7  20.2  20.1  21.0 | 0.3  0.6  0.3  0.8 | 13.4  13.7  13.9  13.8 | 0.2  0.1  0.1  0.3 |
| Wealth index, quintiles‡  Q1  Q2  Q3  Q4 | 4614  2329  1974  1182 | 17.9  18.9  20.2  23.5 | 0.3  0.3  0.4  0.9 | 13.1  13.8  14.2  13.9 | 0.2  0.2  0.2  0.2 |
| Food insecurity in the home  No  Mild  Moderate  Severe | 3469  3572  1889  1166 | 20.1  20.0  19.3  19.2 | 0.6  0.3  0.4  0.5 | 13.8  13.6  13.6  13.8 | 0.1  0.1  0.2  0.3 |
| Urban City  Big cities\|\|  100001 a 1000000 population  0 a 100000 population  Disperse population | 1068  2619  3816  2596 | 21.7  19.9  19.8  17.6 | 0.7  0.3  0.4  0.4 | 14.4  13.8  13.3  13.0 | 0.2  0.2  0.1  0.2 |
| Country region  Central  Atlantic (North)  Oriental  Pacific (West)  Bogotá  Amazonia-Orinoquia | 2416  2099  1972  1215  664  1733 | 18.5  19.3  20.5  18.6  22.4  20.1 | 0.3  0.4  0.4  0.5  1.0  0.6 | 13.7  12.8  13.7  14.1  14.5  13.0 | 0.2  0.2  0.1  0.3  0.3  0.2 |
| *Based on 24-Hour Recall.  †Energy-Adjusted by the density method. Grams/day for every 1000 kcal consumed: 1 kcal/d = 4.18 kJ/d.  ‡According to the WHO.^25^  §The wealth index is a composite measure of a household’s cumulative living standard. The wealth index is calculated using easy-to-collect data on a household’s ownership of selected assets such as televisions and bicycles, materials used for housing construction, type of water supply and sanitation facilities.^21^  \|\|Bogotá, Barranquilla, Medellín, Cali y Bucaramanga. | | | | | |
|  | | | | | |

| **Table 17S** Differences in animal protein intake, g/d for every 1000 kcal consumed, in Colombian population (*18 to 64 years, non-pregnant women*) according to sociodemographic characteristics. National Survey of Nutritional Situation in Colombia (ENSIN, 2015). | | | | |
| --- | --- | --- | --- | --- |
| Variable | Crude difference* (95% CI) | *P*† | Adjusted difference‡ (95% CI) | *P*§ |
| Sex  Males  Females | -  1.1 (0.0, 2.1) | 0.041 | -  0.8 (-0.3, 1.9) | 0.135 |
| Education of head  <5 (Primary or less)  5 to <11  11 to <16  ≥16 (University) | -1.6 (-2.9, -0.3)  -  -0.1 (-1.4, 1.1)  0.7 (-1.2, 2.7) | 0.001 | -0.8 (-1.9, 0.2)  -  -0.5 (-1.9, 0.9)  0.6 (-1.4, 2.5) | 0.341 |
| Wealth index, quintiles\|\|  Q1  Q2  Q3  Q4 | -5.6 (-7.5, -3.6)  -4.6 (-6.6, -2.6)  -3.2 (-5.3, -1.2)  - | <0.0001 | -4.7 (-6.5, -3.0)  -4.3 (-6.1, -2.4)  -3.2 (-5.1, -1.2)  - | <0.0001 |
| Food insecurity  No  Mild  Moderate  Severe | -0.2 (-1.4, -1.0)  -0.8 (-2.1, 0-5)  -0.9 (-2.4, 0.6) | 0.194 | - 1. (-1.1, 1.4)   -0.2 (-1.4, 1.0)  0.2 (-1.2, 1.6) | 0.969 |
| Urban City  Big cities¶  100001 a 1000000 population  0 a 100000 population  Disperse population | -1.8 (-3-3, -0.2)  -1.9 (-3.5, -0.2)  -4.1 (-5.7, -2.5) | <0.0001 | -0.6 (-1.9, 0.7)  0.4 (-0.8, 1.7)  -0.2 (-1.4, 1.1) | 0.776 |
| Country region  Central  Atlantic (North)  Oriental  Pacific (West)  Bogotá  Amazonia-Orinoquia | -  0.7 (-0.4, 1.9)  2.0 (0.9, 3.0)  0.1 (-1.0, 1.3)  4.0 (1.8, 6.1)  1.6 (0.3, 3.0) | 0.001 | -  1.8 (0.6, 3.0)  1.9 (0.9, 2.8)  0.7 (-0.5, 1.8)  3.1 (0.7, 5.4)  2.8 (1.5, 4.1) | 0.007 |
| *Based on 24-Hour Recall. Energy-Adjusted by the density method. Grams/day for every 1000 kcal consumed: 1 kcal/d = 4.18 kJ/d.  Test for linear trend for ordinal predictors. For sex, urbanicity and country region, *P* is from ANOVA. All tests incorporated the complex sampling survey design.  ‡From linear regression models with protein intake as continuous result and indicator variables in the table as predictors except for Height-for-age and BMI-for-age. The estimates for education come from a model that excludes the wealth index and food security, which could be on the causal path. The wealth index estimates excluded food security.  §Adjusted test for linear trend or ANOVA for ordinal or categorical correlates, respectively.  \|\|According to the WHO.^25^  ¶The wealth index is a composite measure of a household’s cumulative living standard. The wealth index is calculated using easy-to-collect data on a household’s ownership of selected assets such as televisions and bicycles, materials used for housing construction, type of water supply and sanitation facilities.^21^  **Bogotá, Barranquilla, Medellín, Cali y Bucaramanga. | | | | |

| **Table 18S** Differences in vegetal (Plants) protein intake, g/d for every 1000 kcal consumed, in Colombian population (*18 to 64 years, non-pregnant women*) according to sociodemographic characteristics. National Survey of Nutritional Situation in Colombia (ENSIN, 2015). | | | | |
| --- | --- | --- | --- | --- |
| Variable | Crude difference* (95% CI) | *P*† | Adjusted difference‡ (95% CI) | *P*§ |
| Sex  Males  Females | -  -0.3 (-0.1, 0.7) | 0.144 | -  0.2 (-0.1, 0.6) | 0.216 |
| Education of head  <5 (Primary or less)  5 to <11  11 to <16  ≥16 (University) | -0.3 (-0.7, 0.1)  -  0.1 (-0.2, 0.5)  0.1 (-0.5, 0.7) | 0.109 | 0.0 (.0.4, 0.4)  -  0.0 (-0.4, 0.4)  0.0 (-0.6, 0.6) | 0.981 |
| Wealth index, quintiles\|\|  Q1  Q2  Q3  Q4 | -0.8 (-1.4, 0.2)  -0.1 (0.6, 0.4)  0.2 (-0.4, 0.9)  - | 0.001 | -0.0 (0.7, 0.6)  0.2 (-0.4, 0.7)  0.3 (-0.3, 1.0)  - | 0.880 |
| Food insecurity  No  Mild  Moderate  Severe | -  -0.3 (-0.6, 0.1)  0.3 (-0.7, 0.2)  -0.0 (-0.7, 0.6) | 0.632 | -  -0.2 (-0.6, 012)  -0.2 (-0.6, 0.3)  0.2 (-0.3, 0.8) | 0.780 |
| Urban City  Big cities¶  100001 a 1000000 population  0 a 100000 population  Disperse population | -  -0.6 (-1.1, -0.0)  -1.1 (-1.6, -0.6)  -1.4 (-2.0, -0.8) | <0.0001 | -  -0.4 (-0.9, 0.2)  -0.9 (-1.4, -0.3)  -1.2 (-1.8, -0.6) | <0.0001 |
| Country region  Central  Atlantic (North)  Oriental  Pacific (West)  Bogotá  Amazonia-Orinoquia | -  -1.0 (-1.5, -0.4)  -0.0 (-0.4, 0.4)  0.3 (-0.3, 1.0)  0.7 (0.1, 1.4)  -0.8 (-1.2, -0.3) | 0.003 | -  -1.0 (-1.5, -0.5)  0.0 (-0.4, 0.5)  0.3 (-0.3, 0.9)  0.0 (-0.7, 0.7)  -0.6 (-1.1, -0.1) | 0.121 |
| *Based on 24-Hour Recall. Energy-Adjusted by the density method. Grams/day for every 1000 kcal consumed: 1 kcal/d = 4.18 kJ/d.  Test for linear trend for ordinal predictors. For sex, urbanicity and country region, *P* is from ANOVA. All tests incorporated the complex sampling survey design.  ‡From linear regression models with protein intake as continuous result and indicator variables in the table as predictors except for Height-for-age and BMI-for-age. The estimates for education come from a model that excludes the wealth index and food security, which could be on the causal path. The wealth index estimates excluded food security.  §Adjusted test for linear trend or ANOVA for ordinal or categorical correlates, respectively.  \|\|According to the WHO.^25^  ¶The wealth index is a composite measure of a household’s cumulative living standard. The wealth index is calculated using easy-to-collect data on a household’s ownership of selected assets such as televisions and bicycles, materials used for housing construction, type of water supply and sanitation facilities.^21^  **Bogotá, Barranquilla, Medellín, Cali y Bucaramanga. | | | | |
